# Beyond Traditional airPLS: Improved Baseline Removal in SERS with Parameter-Focused Optimization and Prediction

**DOI:** 10.1021/acs.analchem.5c01253

**Published:** 2025-07-26

**Authors:** Jiaheng Cui, Xianyan Chen, Yiping Zhao

**Affiliations:** † School of Electrical and Computer Engineering, College of Engineering, 1355 The University of Georgia, Athens, Georgia 30602, United States; ‡ Department of Epidemiology & Biostatistics, College of Public Health, 1355 The University of Georgia, Athens, Georgia 30602, United States; § Department of Physics and Astronomy, 1355 The University of Georgia, Athens, Georgia 30602, United States

## Abstract

Baseline correction is a critical preprocessing step in Raman and surface-enhanced Raman spectroscopy analysis. The adaptive iterative reweighted penalized least-squares (airPLS) method is widely used due to its simplicity and efficiency, but its effectiveness is often hindered by challenges such as baseline smoothness, parameter sensitivity, and inconsistent performance under complex spectral conditions. To address these limitations, we developed an optimized airPLS algorithm (OP-airPLS) that systematically fine-tunes key parameters by using an adaptive grid search method. We further implemented a machine learning model to predict these parameters through spectral shape recognition. A data set of 6000 simulated spectra representing 12 spectral shapes (comprising three peak types and four baseline variations) was used for evaluation. On average, OP-airPLS achieved a percentage improvement (PI) of 96 ± 2%, with the maximum improvement reducing the mean absolute error (MAE) from 0.103 to 5.55 × 10^–4^ (PI = 99.46 ± 0.06%) and the minimum improvement lowering the MAE from 0.061 to 5.68 × 10^–3^ (PI = 91 ± 7%). The optimal parameters for each spectral shape were found to reside within a well-defined linear region in the parameter space. While OP-airPLS significantly improved enhanced baseline correction accuracy, it required substantial computational resources and relied on known true baselines. To overcome these constraints, a machine learning approach combining principal component analysis and random forest (PCA-RF) was developed to directly predict optimal parameters from input spectra. The PCA-RF model demonstrated robust performance and achieved an overall PI of 90 ± 10% while requiring only 0.038 s to process each spectrum. When this method is applied to real spectra, its baseline estimation performance is constrained by both the signal-to-noise ratio and the similarity of the spectral shape to the training data.

## Graphical abstract

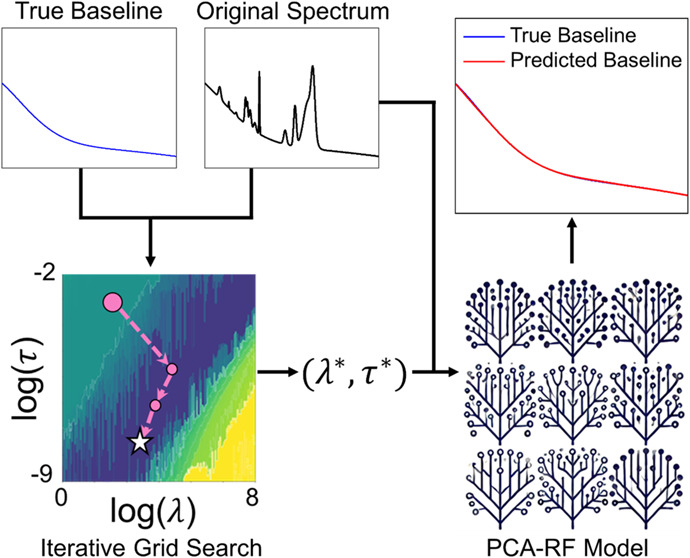

## Introduction

Baseline removal is a crucial preprocessing step in Raman spectroscopy and surface-enhanced Raman spectroscopy (SERS) for complex biological or environmental sample analysis, as it improves the signal-to-noise ratio, reduces background signal interference, and ensures consistency in quantitative analysis. To date, researchers have developed a variety of baseline correction methods to meet these needs, such as polynomial fitting^1–3^ (e.g., Modpoly^1^), iterative fitting^4–10^ (e.g., asymmetric least-squares (ALS)^4^), wavelet transforms^11–14^ (e.g., fully automatic baseline-correction procedure (FABC)^14^), etc. Each of these methods, while widely used, has its limitations. Polynomial fitting can introduce artificial bumps in featureless regions and requires tuning the polynomial order, often leading to suboptimal results. Iterative fitting methods, although efficient, lack accuracy for complex spectral features. Wavelet transforms can handle localized spectral features well but struggle with spectral complexity and variability.

Among these, the adaptive iterative reweighted penalized least-squares (airPLS) method is known for its simplicity and efficiency.^10^ The original airPLS paper compared its performance with FABC^14^ and ALS^4^ in two independent experiments. The first used simulated Raman spectra of three distinguished Gaussian peaks combined with linear or Gaussian baselines and assessed how well airPLS, FABC, and ALS predicted peak heights. For the linear baseline, the heights of two of the three peaks predicted by airPLS were closer to the true values than those by FABC and ALS. For the Gaussian baseline, airPLS was more accurate than FABC and ALS for the smaller peak, while for the two larger peaks, airPLS was considered comparable. The second experiment aimed to predict the concentrations of methanol, acetonitrile, and distilled water in mixtures based on Raman spectra. A partial least-squares regression algorithm was applied to the baseline-corrected spectra. airPLS achieved marginally better coefficient of determination (*R* ^2^) and root-mean-square error (RMSE) than those predicted via FABC and ALS.

The airPLS algorithm predicts baselines by iteratively optimizing a loss function that balances two competing objectives (see Section S1 of Supporting Information (SI) for a detailed description): fidelity (ensuring the predicted baseline closely approximates the observed spectrum) and smoothness (penalizing rapid fluctuations in the baseline). The algorithm employs three key parameters that govern this optimization process: λ (penalizing smoothness), τ (convergence tolerance), and *p* (smoothness order). λ controls the relative weight given to smoothness versus fidelity in the loss function with larger λ values producing smoother baselines. *p* determines the mathematical order of smoothness constraints applied to the baseline, where higher values enforce greater continuity in baseline derivatives. τ defines the stopping criteria for the iterative optimization process with smaller τ values requiring tighter convergence before termination. The default values used in ref^10^ are λ = 100, τ = 0.001, and *p* = 1. With these default parameters (referred to as DP-airPLS), three issues typically arise as shown in Figure S2: (1) nonsmooth, piecewise linear baselines, (2) significant errors in broad peak regions leading to large mean absolute errors (MAE, defined in eq S8), and (3) difficulties with complex spectral regions. This comparison demonstrates the need for improved airPLS parameter optimization.

Several modified airPLS versions addressed specific limitations of the original algorithm. IAsLS (improved asymmetric least-squares)^7^ incorporated second-order differentiation to improve baseline smoothness and achieved lower RMSE on simulated spectra with exponential and Gaussian baselines, but lacked systematic parameter tuning. IarPLS (iteratively adaptive reweighted penalized least-squares)^8^ and IairPLS (improved adaptive iterative weighted penalized least-squares)^9^ modified the reweighting process to better handle peak regions—IarPLS using inverse square root weighting and IairPLS employing exponential weighting functions—both demonstrating improved RMSE across different noise levels. However, these approaches share common limitations: inadequate baseline smoothness control, lack of comprehensive parameter optimization (particularly τ tuning), and limited evaluation of complex spectral scenarios. None systematically addressed the interaction between λ, τ, and *p* parameters, which can lead to artifacts such as negative corrected spectral values when parameter selection remains suboptimal.

To address the aforementioned issues, here, an adaptive grid search optimization algorithm (optimized airPLS, abbreviated as OP-airPLS) is developed to optimize parameter selection in airPLS. By fixing *p* = 2 and systematically adjusting λ and τ values, this algorithm can produce smooth baselines and minimize the MAE across 12 simulated spectral shapes. On average, the algorithm has achieved a percentage improvement (PI) of 96 ± 2% over DP-airPLS. Based on the predicted optimal λ and τ values, we construct a machine learning model—ML-airPLS—combining principal component analysis and random forest (PCA-RF) to directly predict optimal λ and τ values from input spectra. This approach aligns with recent advances in using artificial intelligence applications for SERS analysis.^15,16^ Using the PCA-RF model, we achieved PI exceeding 70 in 90% of cases, with the PI further improving to 90 ± 10% after outlier removal. To further implement the above proposed strategy to real measured spectra, we need to include more spectral shapes in the training set and systematically explore the effect of noise in spectra. Overall, the ML-airPLS offers a robust and generalizable solution for the spectral baseline correction.

## Methods

To address the limitations of DP-airPLS, we developed a two-stage approach that combined systematic parameter optimization with machine learning prediction. First, we implemented an adaptive grid search algorithm OP-airPLS to identify optimal (λ, τ) parameter combinations across diverse synthetic spectral conditions. Second, we trained a machine learning model (ML-airPLS) to predict these optimal parameters directly from spectral features, eliminating the computational burden and true baseline requirement of iterative optimization.

### Iterative Grid Search Framework for Optimizing airPLS Parameters

To systematically explore the (λ, τ) parameter space for fixed *p* = 2 and identify optimal combinations, we implemented a comprehensive grid search algorithm, OP-airPLS. The algorithm evaluates parameter combinations across predefined ranges of λ and τ values, systematically testing each combination and selecting the one that minimizes MAE between predicted and true baselines for each spectrum. This optimization framework is specifically designed for experiments where the true baseline is known (e.g., simulated spectra), enabling objective performance evaluation based on quantitative error metrics. The algorithm uses an adaptive grid refinement approach that progressively searches finer parameter regions around the best-performing combinations. Convergence is determined when the MAE improvement becomes negligible (less than 5% change) across 5 consecutive refinement steps, indicating that further parameter adjustment yields diminishing returns in baseline correction accuracy. The complete algorithm flowchart is presented in Figure S3, with a detailed description of the 12 algorithmic steps provided in Section S3.

For practical implementation, we evaluated the grid search performance using synthetic data sets comprising 12 spectral shapes, with 500 spectra per shape (detailed in the following section). The optimization algorithm was applied sequentially to all 500 spectra within each spectral shape group. For the first spectrum in each group, we initialized (λ_0_, τ_0_) = (100, 0.001) (the default values in DP-airPLS). For subsequent spectra in the same group, we initialized (λ_0_, τ_0_) to be (λ*, τ*) of the optimized parameters from the previous spectrum, leveraging the similarity of optimal parameters within the same spectral shape group.

To quantify the improvement of OP-airPLS over DP-airPLS, we define the percentage improvement (PI) based on the mean absolute errors obtained by each method

1 $$PI\;\left(\%\right)=\frac{\left|MAE_{OP}-MAE_{DP}\right|}{MAE_{DP}}\times 100\%$$

where MAE_DP_ represents the MAE between the true baseline and the predicted baseline using DP-airPLS parameters, and MAE_OP_ represents the MAE between the true baseline and the predicted baseline using OP-airPLS optimized parameters (λ*, τ*). According to eq 1, a PI > 90% is equivalent to a reduction of the MAE by 1 order of magnitude or more, and a PI > 99% is equivalent to a reduction by 2 orders of magnitude.

The OP-airPLS algorithm was implemented in Python 3.11.5, using key libraries including NumPy 1.24.3,^17^ Pandas 2.2.1,^18^ Matplotlib 3.8.4,^19^ SciPy 1.11.1,^20^ and Scikit-learn 1.4.1.^21^ Our implementation includes several critical improvements have been made to the original DP-airPLS code, including enhanced numerical stability and overflow prevention, as detailed in Section S4. All calculations were performed on a workstation equipped with an Intel Core i7–13700KF CPU (3.40 GHz) and 64 GB RAM.

### Synthetic Spectral Data Set Construction

The detailed procedure of synthetic spectral data set construction is described in Section S5. To comprehensively evaluate the grid search algorithm’s performance across diverse spectroscopic conditions, based on our experience in chemical and biological detections using SERS,^22^ we identified three prevalent peak shapes and four characteristic baseline shapes. The three peak shapes are broad (B), convoluted (C), and distinct (D), representing different degrees of peak overlap. The four baseline shapes are exponential (E), Gaussian (G), fifth-order polynomial (P), and sigmoidal (S). This systematic notation allows us to clearly identify all 12 possible spectral shapes. Each spectral shape is denoted by connecting the peak shape and baseline shape abbreviations with “&”—for example, B&E represents a spectrum with broad peak shape and exponential baseline. All spectra along with their corresponding baselines are presented in Figure S5.

### Machine Learning Model Design

For ML-based airPLS parameter prediction, we used the generated data set of 6000 simulated spectra as inputs, with their corresponding optimal parameters (λ*, τ*) as target outputs. To ensure balanced representation across spectral shapes, we applied stratified sampling and split the data set into training, validation, and testing sets in an 8:1:1 ratio, resulting in 400, 50, and 50 spectra per spectral shape, respectively. To maintain consistency across all ML model evaluations, we fixed the random number seed.

We evaluated multiple ML models for predicting (λ*, τ*) values, including Random Forest (RF),^23^ Extreme Gradient Boosting (XGBoost),^24^ Long Short-Term Memory (LSTM),^25^ Deep Neural Network (DNN),^26^ Convolutional Neural Network (CNN). All ML model configurations can be found in Section S6. To identify the best-performing models, we established a two-stage selection process using PI. We first defined two key performance metrics for each spectral shape group

2 $$\{\begin{array}{l} \mathrm{Pct}\left(PI_{\mathrm{ML}}\geq 70\%\right)=\frac{\#\left(spectra\;achieving\;PI_{\mathrm{ML}}\geq 70\%\right)}{\#\left(all\;spectra\right)}\times 100\%\\ \mathrm{Pct}\left(PI_{\mathrm{ML}}\geq 80\%\right)=\frac{\#\left(spectra\;achieving\;PI_{\mathrm{ML}}\geq 80\%\right)}{\#\left(all\;spectra\right)}\times 100\% \end{array}$$

In the first selection round, we counted how many spectral shapes (out of 12) each model could successfully process, with success defined as achieving both *Pct*(PI_*ML*_ ≥ 70%) ≥ 70% (minimum requirement) and *Pct*(PI*_ML_* ≥ 80%) ≥ 80% (higher target). In the second round, among the top-performing models, we selected the configuration with the highest average performance across all spectral shapes.

## Results and Discussion

### Impact of λ and τ in airPLS Baseline Prediction

When *p* is fixed to 2, baseline fitting by airPLS strongly depends on the (λ, τ) values. The red curve in Figure 1A is the predicted baseline-removed spectrum *I*_pred_ using the same values (λ = 10^2^, τ = 10^–3^) in DP-airPLS, and it is compared with the true spectrum *I*_true_ (black curve). *I*_pred_ deviates significantly from *I*_true_, particularly in the wavenumber regions of 457–553, 800–985, and 1270–1920 cm^–1^, resulting in a very high MAE of 48.6. For comparison, the MAE_DP_ is 10.6, which is significantly lower than 48.6. This significant difference in MAE highlights that simply changing *p* from 1 to 2 with a fixed (λ, τ) yields a worse predicted baseline, which implies that both λ and τ need to be tuned simultaneously.

**Figure 1:**
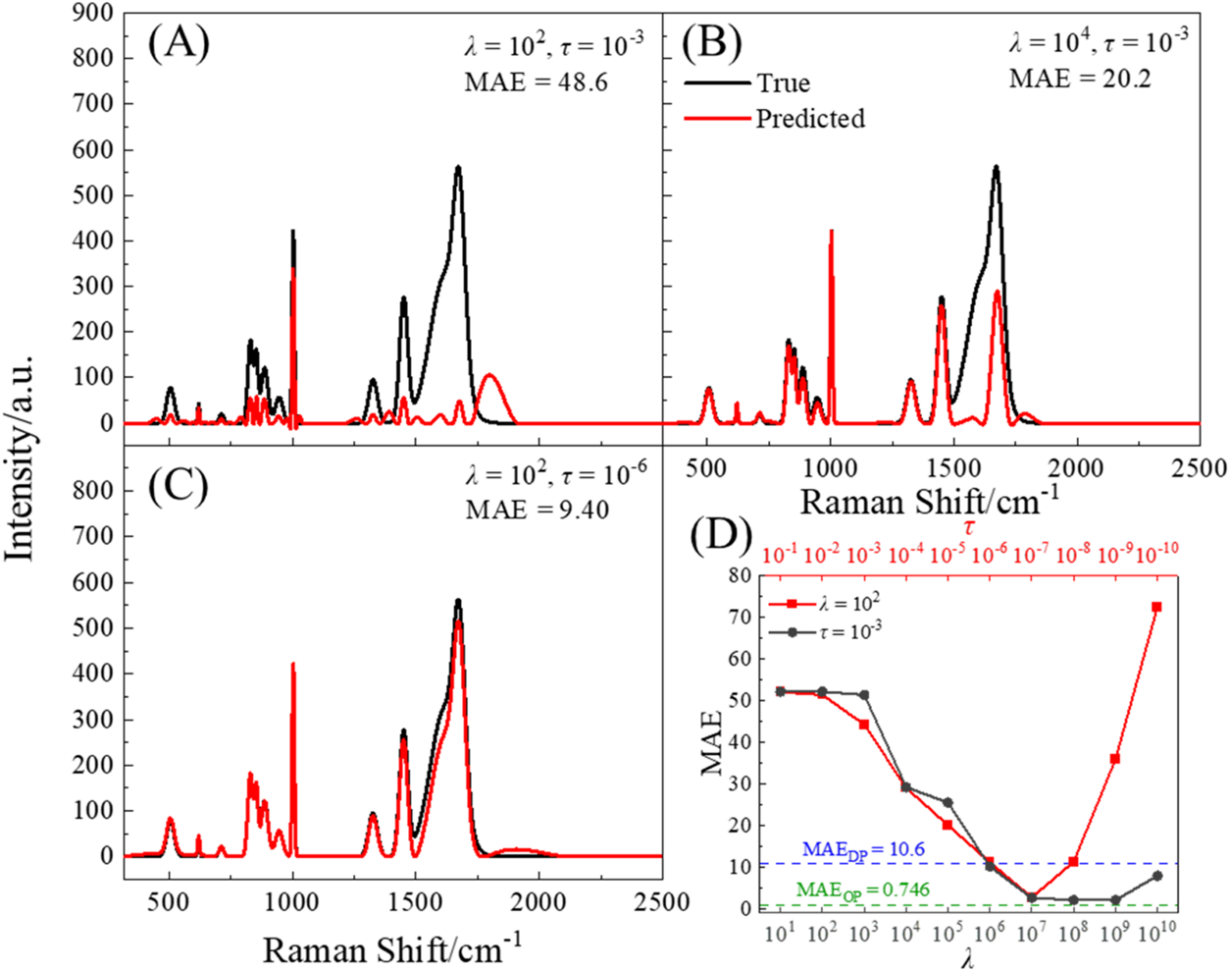
AirPLS baseline-removed spectrum for different parameter settings: (A) λ = 10^2^, τ = 10^–3^; (B) λ = 10^4^, τ = 10^–3^; and (C) λ = 10^2^, τ = 10^–6^. The black curves represent the true spectrum, while the red curves show the airPLS-estimated spectrum. (D) MAE between the true spectrum and the airPLS-estimated spectrum as a function of τ (λ = 10^2^, red curve) and λ (τ = 10^–3^, black curve), respectively. The MAE for DF-airPLS is 10.6 (blue dashed line), while the MAE for OP-airPLS is 0.746 (green dashed line).

To investigate the effect of λ and τ, we independently vary each parameter systematically. When λ increases from 10^2^ to 10^4^ while remaining at τ = 10^–3^, as shown in Figure 1B, though the *I*_pred_ still deviates significantly from *I*_true_ in 1500–1920 cm^–1^, it is closer to the true spectrum in 457–553, 800–985, and 1280–1500 cm^–1^ regions. The resulting MAE is 27.2 compared to 48.6 in Figure 1A. This is because as λ increases, the penalizing term in the loss function becomes more important, leading to a smoother and better baseline while discouraging the fitted baseline to be close to the original spectrum. When τ is reduced from 10^–3^ to 10^–6^ while keeping λ = 10^2^, as shown in Figure 1C, the resulted *I*_pred_ is significantly improved, especially in the 457–553, 800–985, and 1270–1920 cm^–1^ regions. Though *I*_pred_ and *I*_true_ still have small visible differences in 1500–2000 cm^–1^, the MAE has reduced to 9.40, which is slightly smaller than MAE_DP_. According to the definition of τ, decreasing τ tightens the convergence criteria, forcing the iterative updates of the weight matrix to converge in more iterations, e.g., from 13 (Figure 1A) to 46 (Figure 1C). It reduces the portion of the baseline removal spectrum that has negative intensities, which is more in line with the true spectrum.

The effects of systematically tuning λ and τ are summarized in Figure 1D, where MAE­(*I*_pred_(Δν), *I*_true_(Δν)) is plotted against λ and τ. When fixing τ = 10^–3^ (black curve), MAE monotonically decreases from 48.7 to 1.83 when λ increases from 10^1^ to 10^7^, and then slightly increases when λ increases from 10^7^ to 10^10^. When λ = 10^2^ (red curve), the MAE-τ curve mimics a parabola, reaching a minimum of 2.42 at τ = 10^–7^. The minimum MAEs in both cases are significantly smaller than MAE_*DP*_, as indicated by the blue dashed line in Figure 1D. These results clearly demonstrate that both λ and τ need to be optimized in order to obtain a better baseline fitting. In fact, by simultaneously adjusting λ and τ to 1.53 × 10^5^ and 9.65 × 10^–6^, we later find a minimum MAE of 0.746 (indicated by the green dashed line in Figure 1D), significantly smaller than MAE_DP_. Thus, for any spectrum with different spectral shapes, one shall simultaneously tune λ and τ to the optimal parameter set, denoted as (λ* and τ*), and obtain a minimum MAE.

### Performance of Grid Search

Given the critical importance of simultaneous λ and τ optimization demonstrated above, we applied our iterative grid search methodology (OP-airPLS) described in Methods section to automatically identify optimal parameter combinations across diverse spectral conditions. To illustrate the effectiveness of the proposed optimization algorithm, we compare the baselines calculated using OP-airPLS and DP-airPLS for the same spectrum shown in Figures S2 and 1, which represents a B&E baseline (details see Section S7). Figure S6A shows the extracted baseline (red curve) obtained using OP-airPLS with (λ*, τ*) = (1.53 × 10^5^, 9.65 × 10^–6^). The resulting baseline is smooth and closely matches the true baseline. Figure S6D–F show three additional representative spectral shapes with their corresponding optimized baseline fittings (red curves): C&G (Figure S6D), C&S (Figure S6E), and D&P (Figure S6F). The optimized parameters (λ*, τ*) for these cases are (2.14 × 10^3^, 5.23 × 10^–7^), (5.86 × 10^5^, 4.03 × 10^–5^), and (6.85 × 10^1^, 3.16 × 10^–8^), respectively. In all cases, the fitted baselines are visually smooth and match well with the true baselines. The MAE_OP_ are 1.04 × 10^–3^, 1.37 × 10^–3^, and 6.56 × 10^–4^, while the corresponding MAE_DP_ are significantly higher at 3.02 × 10^–2^, 3.15 × 10^–2^, and 7.71 × 10^–2^. This optimization process reduces MAE by approximately one-2 orders of magnitude. The results of Figure S6 demonstrate that the proposed optimization successfully addresses the three critical issues of the DP-airPLS outlined in the Introduction section: nonsmooth baselines, large MAE, and distortions in peak regions. To quantify the improvements of MAE_OP_ over MAE_DP_, we calculated the PI defined in Methods section. The calculated PIs for the cases in Figures S6A,D,E, and S6F are 93.0, 96.6, 95.6, and 99.1%, respectively. These high PI values highlight the significant reduction in MAE achieved by OP-airPLS. These findings reaffirm the algorithm’s capacity to provide reliable baseline fitting, even under challenging spectral conditions.

Detailed convergence analysis (Section S8) reveals that the grid search algorithm converges to local rather than global minima in the parameter landscape. Despite converging to different local minima depending on starting points, the algorithm achieves consistently high performance across these minima (PI > 90% in most cases) with rapid convergence averaging 4 ± 2 iterations.

### Evaluation of OP-airPLS on Baseline Removal Improvement

To comprehensively evaluate the optimization algorithm, we applied OP-airPLS to all 6000 spectra (500 spectra × 12 spectral shapes). Figure 2A summarizes the distribution of (λ*, τ*) values for 10 selective spectra from each of the 12 spectral shapes in the log­(τ)–log­(λ) plane. For each spectral shape, the λ* and τ* points form tight clusters. The dashed ovals in Figure 2A plot the 95% confidence ellipse^27^ derived from all 500 (λ*, τ*) values for three selected spectral shapes: B&E (green), B&P (gray), and C&E (blue). The confidence ellipses for B&E and C&E are relatively small, while the B&P ellipse is noticeably larger, demonstrating that the distribution of optimized (λ*, τ*) values depend strongly on the spectral shape. Across all 12 spectral shapes, the optimized (λ*, τ*) values consistently fall within a distinct diagonal valley region. To formally define this region, an overall “common small MAE region” *R*_overall_ (the background mapping and red ellipse in Figure 2A) was derived based on the convergence patterns shown in Section S9, characterized using a linear equation

3 $$\log\left(\tau^{*}\right)=0.865\log\left(\lambda^{*}\right)-8.765,\log\left(\lambda^{*}\right)\in \left[0.980,4.571\right]$$

with a high goodness-of-fit (*R* ^2^ = 0.956). This result highlights the stability of the optimization algorithm in consistently converging to suitable local minima, even under varying spectral conditions. In addition, eq 3 also provides practical guidance for initializing parameters across different spectral shapes.

**Figure 2:**
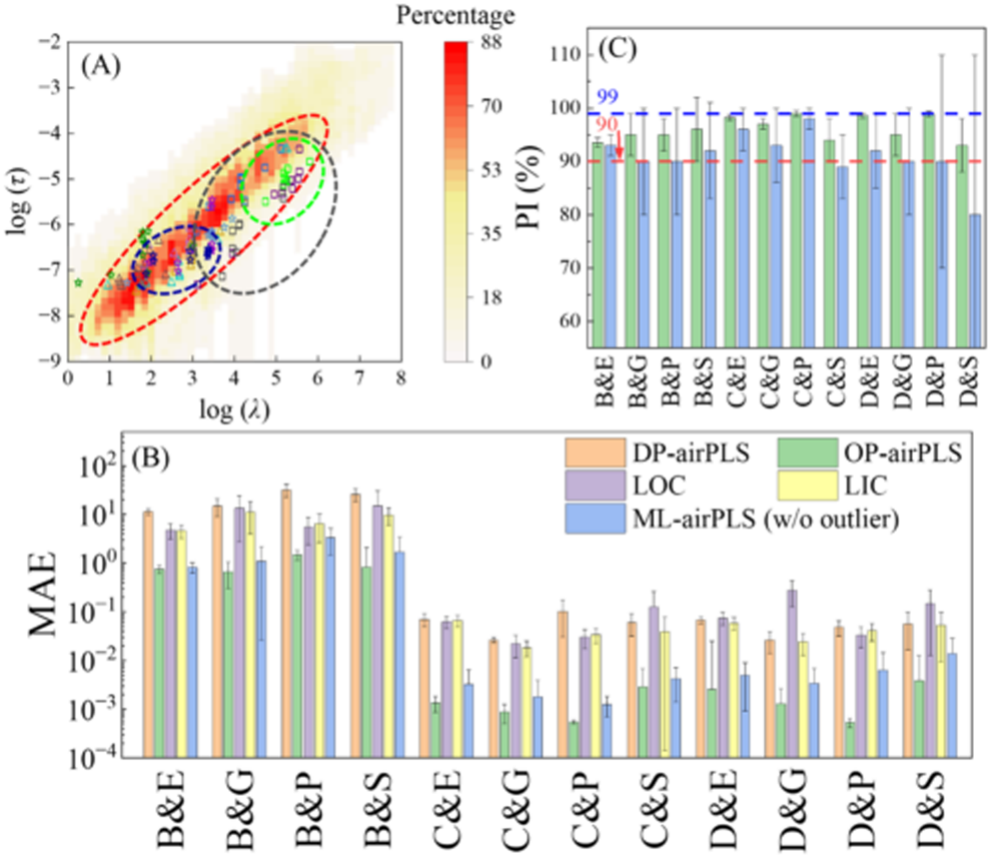
(A) Distribution of selected (λ*, τ*) values for 12 spectral shapes is plotted in log–log scale, with 10 example spectra per shape. Squares represent broad peaks; triangles denote convoluted peaks, and stars indicate distinct peaks: Green square: **B&E**, Blue square: **B&G**, Gray square: **B&P**, Violet square: **B&S**, Dark blue star: **C&E**, Silver star: **C&G**, Purple star: **C&P**, Dark green star: **C&S**, Yellow triangle: **D&E**, Electric blue triangle: **D&G**, Light purple triangle: **D&P**, Brown triangle: **D&S**. The red ellipse outlines the overall (λ*, τ*) distribution. The 95% confidence ellipses for B&E (green), B&P (gray), and C&E (blue) are shown. A heatmap in the background represents the optimal region *R*_overall_ across the 12 spectral shapes. (B) Bar plot of the average and standard deviation of MAE values for different baseline estimation methods across the 12 spectral shapes: MAE_DP_ (orange), MAE_OP_ (green), MAE_LOC_ (purple), MAE_LIC_ (yellow), and MAE_ML_ (blue). (C) Bar plot of the average PI and standard deviation of OP-airPLS (green) and ML-airPLS (blue) for 12 spectral shapes. The red horizontal dashed line indicates a 90% PI, while the blue horizontal dashed line represents a 99% PI.

Given this distinct clustering pattern, we investigated whether an averaged parameter set could serve as a practical alternative to replace those by DP-airPLS. Two simple parameter selection strategies were evaluated: logarithmic center (LOC) and linear center (LIC). The detailed analysis is presented in Sections S10. Corresponding MAE and PI for LOC and LIC can be found in Table S7, and the conclusion is that although either LOC or LIC could improve baseline estimation performance for some spectral shapes, it fails for some others; i.e., there are no default optimal parameter sets for airPLS baseline estimation.

Figure 2B summarizes the average MAE values and their standard deviations for different methods across all spectral shapes: MAE_DP_ (orange bars), MAE_OP_ (green bars), MAE_LOC_ (purple), and MAE_LIC_ (yellow). The average MAE_DP_ varies between 11.4 and 31.8 for peak shape B, while for peak shapes C and D, it ranges from 0.030 to 0.100. This difference arises because peak shape B exhibits larger intensity scales than peak shapes C and D. After optimization, the average MAE_OP_ is significantly reduced, ranging from 0.520 to 1.520 for peak shape B and from 4.85 × 10^–4^ to 5.68 × 10^–3^ for peak shapes C and D. The greatest improvement is observed for C&P, where the average MAE decreases from 0.103 to 5.55 × 10^–4^. The smallest improvement happens at C&S, with the average MAE decreasing from 0.061 to 5.68 × 10^–3^. Despite being the smallest improvement, this still corresponds to a reduction of more than 1 order of magnitude, underlining the importance of considering both peak arrangement and baseline shape variations in future baseline correction studies.

Figure 2C plots the PI values for each spectral shape. The highest PI is observed in C&P, with an average of 99.0 ± 0.5%, while the lowest is 93.5 ± 0.9% for C&S. On average, the optimization algorithm achieves a PI of 96 ± 2%. Notably, for C&P, the PI consistently exceeds 99% (blue horizontal line), indicating a two-order-of-magnitude reduction in MAE—an excellent performance level. More generally, all spectral shapes yield an average PI above 90% (red horizontal line), confirming that the optimization algorithm successfully enhances baseline correction across a wide range of spectral conditions. Furthermore, the small standard deviations relative to their corresponding mean values highlight the stability and reliability of the optimization process across different spectral shapes.

### Machine Learning Parameter Prediction

While OP-airPLS demonstrates excellent performance across all spectral shapes, three critical limitations prevent its practical application to real experimental data. First, the iterative optimization requires computationally intensive grid search, averaging 80 s per spectrum. Second, experimental spectra lack known true baselines, making it impossible to calculate the MAE needed for optimization. Third, simple parameter averaging strategies (LOC and LIC) fail to provide reliable improvements. To overcome these challenges, we propose using an ML approach (ML-airPLS) to predict optimal (λ* and τ*) values directly from input spectra. Unlike simple averaging methods, ML models can learn complex, nonlinear relationships between spectral patterns and optimal parameters, making them well-suited for this task.

To demonstrate the effectiveness of ML approaches, Figure 3A–D show representative examples of the PCA-RF model’s performance by comparing ML-predicted baselines (green curves) with OP-airPLS (red curves) and true baselines (purple curves) for four representative spectra from Figure S7: B&E (Figure 3A), C&G (Figure 3B), C&S (Figure 3C), and D&P (Figure 3D). Visually, the ML-airPLS-predicted baselines are smooth and closely align with both the OP-airPLS and the true baselines. The corresponding MAE_ML_ values for these spectra are 0.797, 1.45 × 10^–3^, 5.36 × 10^–3^, and 6.98 × 10^–4^, respectively, comparing to MAE_OP_ values of 0.746, 1.04 × 10^–3^, 1.37 × 10^–3^, and 6.56 × 10^–4^. The calculated PI_ML_ values are 92.4, 95.2, 83.0, and 99.1%, respectively. These high PI values highlight a significant reduction in MAE compared to DP-airPLS and reaffirm the effectiveness of ML approaches in providing reliable baseline correction even for challenging spectral conditions.

**Figure 3:**
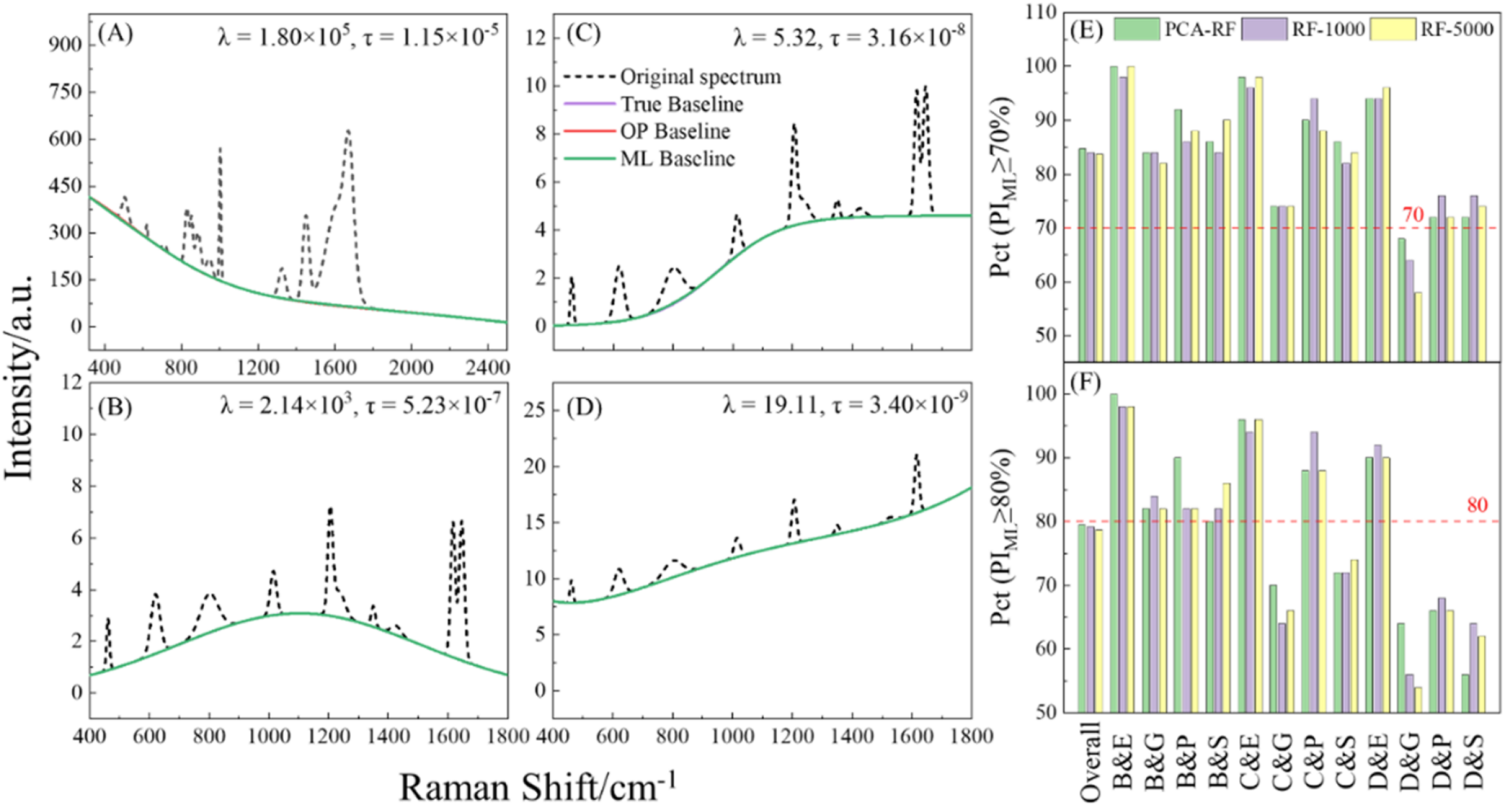
Comparison of the PCA-RF predicted baseline (green curves), OP-airPLS-estimated baseline (red curve), and the true baseline (purple curve) for a representative (A) B&E, (B) C&G, (C) C&S, and (D) D&P spectrum. Comparison of the percentage of spectra achieving (E) PI_ML_ ≥ 70% and (F) PI_ML_ ≥ 80% from three ML models, PCA-RF (green bars), RF-1000 (purple bars), and RF-5000­(yellow bars), across different spectral shapes.

Following the two-stage selection process described in the Methods section, performance metrics for all ML models are obtained and summarized in Figure S11, arranged in descending order by performance. Three models emerged as top performers: PCA-RF with 100 trees (abbreviated as PCA-RF), RF with 1000 trees (RF-1000), and RF with 5000 trees (RF-5000), each successfully processing 11 out of 12 spectral shapes at the 70% threshold and 7 out of 12 at the 80% threshold (Table S6). Among these candidates, PCA-RF demonstrated the best overall performance. At the 70% PI threshold (Figure 3E), PCA-RF achieved an 84.7% success rate, slightly outperforming RF-1000 (84.0%) and RF-5000 (83.7%). At the 80% threshold (Figure 3F), PCA-RF maintained a 79.5% success rate compared with 79.2% for RF-1000 and 78.7% for RF-5000. When comparing which model achieved the highest *Pct*(PI_ML_ ≥ 80%) for each spectral shape, PCA-RF and RF-1000 ranked first (tied) in 5 shapes and RF-5000 only in 3 shapes. This head-to-head comparison across individual spectral shapes, combined with its slightly higher average performance, establishes PCA-RF as the optimal ML-airPLS model for baseline correction. Detailed performance statistics, including MAE and PI values (both with and without outliers), computational time, and outlier counts across spectral shapes of PCA-RF, are summarized in Table S7.

While the PCA-RF consistently delivers high-quality predictions, occasional deviations from optimal parameters occur. These outlier cases, defined in Section S11, account for only 10% of predictions (row 8 of Table S7). After excluding outliers, PCA-RF achieved an average PI_ML_ of 90 ± 10%, reducing MAE by 1 order of magnitude compared to DP-airPLS. As shown in Figures 2B,C, PCA-RF outperformed DP-airPLS, LOC, and LIC across all 12 spectral shapes, with particularly strong performances in B&E (PI_ML_ = 93 ± 2% with no outliers) and C&P (PI_ML_ = 90 ± 10% with no outliers). Even for the least improved spectral shape, D&S, the average PI_ML_ remained at 80% after outlier removal.

In terms of computational efficiency, PCA-RF processed each spectrum in 0.038 s on average using parallel processing (by setting the “*n_jobs*” parameter to −1 during model training and inference), making it comparable to DP-airPLS (0.013 s) and significantly faster than OP-airPLS (80 s). This represents a ∼2100× speedup over iterative optimization, with batch processing demonstrating near-linear scalability across varying data set sizes. The model’s robust performance across diverse spectral patterns, combined with its computational efficiency, demonstrates its potential for real-world applications. This approach will be incorporated into our group’s free online spectroscopy processing tool, SpectraGuru.^28^

### Robustness Analysis of ML-airPLS for Complex Shape and Noisy Spectrum Scenarios

Practically the real baseline and spectral shapes could be more complex than the 12 proposed 12 spectral shapes. To evaluate the ML-airPLS (i.e., PCA-RF) performance on realistic spectral complexity, we systematically constructed some compound spectra by adding two baselines with equal weighting: E + P, E + G, E + S, and G + S, plus a compound peak shape (B + C) (details see Section S12) with a total of 12 compound spectra being generated. We applied the previously trained PCA-RF model to predict the (λ* and τ*) values for these spectra. As shown in Figure S13 and Table S9, the PCA-RF model demonstrated robust performance, achieving PI > 50% for all 12 compound shapes and PI > 90% for 7 spectra. Notably, all spectra with E + S compound baselines achieved PI > 90%, and the compound peak shape (B + C) consistently met our 70% PI threshold. These results confirm that ML-airPLS can handle realistic spectral complexity that extends beyond the proposed categories used in the model training.

Real BPE and CoV229E SERS spectra were also used to estimate the performance of the ML-airPLS (Section S13). Here, the baseline-removed spectra by WiRE software (Renishaw, which provides expert-validated baseline correction algorithms) were used as our reference standard for baseline validation. Direct application of ML-airPLS to experimental spectra without preprocessing yielded consistently negative PI values. We hypothesized that spectroscopic noise was the primary limitation, as the PCA-RF model was trained on noise-free synthetic data. Based on this hypothesis, we conducted comprehensive noise sensitivity analysis, as detailed in Section S13. The synthetic data sets were augmented with Gaussian noise at 10 different signal-to-noise ratio (SNR) levels, ranging from 6.47 to 49.92 based on experimental distributions from our experimentally obtained BPE and CoV229E data sets, with a total of 300,000 noisy spectra. Our preliminary investigations confirmed that PCA-RF performance degraded significantly with noise, with ML-predicted parameters systematically deviating from optimal values as SNR decreased. Even at the highest experimental SNR (49.92), the model achieved negative PI values across all spectral shapes, indicating that airPLS inherently conflicts with noise-induced fluctuations.

Therefore, for real spectrum baseline prediction, a straightforward method to improve airPLS estimation is to increase spectral SNR. Based on our noise sensitivity findings, we applied Savitzky-Golay denoising (window length = 15, order = 2)^29^ to enhance experimental spectra SNR above 100 before ML-airPLS processing as detailed in Section S14. Here, the baseline-removed spectra by WiRE software (Renishaw, which provides expert-validated baseline correction algorithms) were used as our reference standard for baseline validation. The final performance varied dramatically: the CoV229E spectra achieved PI values up to 67.3% with smooth baseline predictions (Figure 4A), while BPE spectra still consistently showed negative PI values with ML baselines strongly deviating from the WiRE reference (Figure 4B). Figure 4C shows the overall performance distribution across both data sets through box plots. To understand this performance difference, we calculated cosine similarity (defined in eq S18) between experimental spectra and our training data from 12 spectral shapes, revealing that CoV229E spectra showed high similarity to the B&P spectral shape (0.933 ± 0.067), while BPE spectra exhibited poor similarity to all training combinations, with maximum similarity of only 0.540 ± 0.039 to the C&E spectral shape, confirming that ML-airPLS effectiveness depends strongly on spectral similarity to training data.

**Figure 4:**
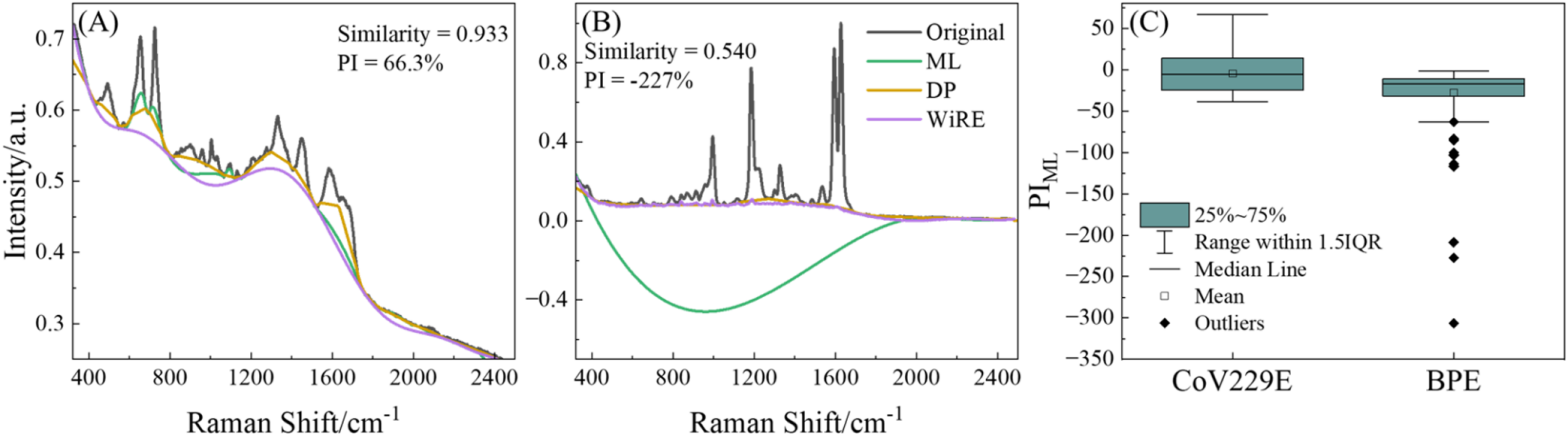
Baseline-correction performance of ML-airPLS on denoised experimental SERS spectra. (A) CoV229E spectrum (black; SNR = 106) with WiRE-derived baseline (purple), ML-optimized baseline (green), and DP baseline (brown); the ML-optimized baseline is smoother and more aligned with the WiRE baseline than the DP baseline. (B) BPE spectrum (black; SNR = 357) with corresponding baselines; the ML baseline deviates markedly from the WiRE result, whereas the DP baseline closely follows it. (C) Box plots of PI for BPE and CoV229E data sets; CoV229E has a mean PI near zero, while all BPE samples exhibit negative PI values.

These findings reveal current limitations in ML-airPLS generalizability and indicate three potential research directions for future development: (1) developing noise-robust approaches through either recalculating the OP parameters for every noisy spectrum or optimizing the current denoising algorithm and parameters; (2) expanding synthetic training data sets to encompass broader baseline and peak shape variations representative of diverse experimental conditions; (3) developing quantitative methods to evaluate baseline removal performance for experimental spectra without known true baselines.

## Conclusions

The default parameter selection in airPLS (DP-airPLS) faces three main challenges. To address these issues, we developed OP-airPLS, an optimization algorithm that fixes *p* = 2 and systematically tunes the remaining two parameters (λ and τ) to minimize the MAE of baseline fitting. This optimization approach consistently converges to local minima and achieves significant improvements over DP-airPLS. The OP-airPLS was evaluated across 12 different spectral shapes, incorporating three different peak shapes and four baseline types, with 500 spectra per category. The results showed that OP-airPLS consistently reduced MAE, achieving an average improvement of 96 ± 2%, typically lowering errors by at least 1 order of magnitude compared to that of DP-airPLS. The most significant improvement was observed for the C&P spectral shape, where the MAE decreased from 0.103 to 5.55 × 10^–4^, yielding a PI of 99.0 ± 0.5%. The least improvement occurred for the C&S spectral shape, where the MAE reduced from 0.061 to 5.68 × 10^–3^, corresponding to a PI of 93.5 ± 0.9%. Compared with DP-airPLS, OP-airPLS effectively mitigates large MAEs, particularly in cases with complex peak spectral shapes.

While OP-airPLS significantly enhances baseline correction accuracy, it introduces two new challenges: long computational time and the need for a true baseline to optimize (λ, τ). To overcome these limitations, we developed an ML model, PCA-RF, which directly predicts optimal parameters (λ* and τ*) from input spectra. This PCA-RF model demonstrated robust performance across all spectral shapes, achieving PI_ML_ > 70% in 11 out of 12 spectral shapes and PI_ML_ > 80% in 7 out of 12 shapes. Among the 50 test spectra per spectral shape, an average of 5 spectra were identified as outliers. After removing these outliers, PCA-RF achieved an overall PI_ML_ of 90 ± 10%, effectively reducing MAE by an order of magnitude compared to DP-airPLS while also dramatically improving computational efficiency—reducing the processing time from 80 s to just 0.038 s per spectrum. Testing on compound spectral shapes combining multiple baseline trends and peak characteristics confirmed effectiveness beyond discrete training categories, achieving PI > 50% for all compound scenarios, and PI > 90% for 7 out of the 12 compound spectra.

Despite these advancements, several challenges remain for future research, including noise sensitivity, limited generalizability to diverse baseline shapes, and the need for more advanced models to reduce outlier rates. Future work should focus on developing noise-robust approaches, expanding synthetic training data sets, and creating quantitative evaluation methods for experimental spectra. Nevertheless, the PCA-RF model represents a significant step forward in baseline correction, offering a computationally efficient, data-driven approach that eliminates both the need for true baselines and the computational burden of iterative optimization. With its upcoming integration into SpectraGuru, this improved approach will be freely accessible to the broader spectroscopy community, providing a valuable tool for Raman/SERS spectral analysis and real-world applications within appropriate scope conditions and where true baselines are unavailable.

## Supplementary Material



## Data Availability

The code and data used in this manuscript are available at https://github.com/jimcui3/OP-airPLS.

## Notes

Including additional information on DP-airPLS, issues with DP-airPLS, steps of the OP-airPLS algorithm, improvements to the original official Python code, simulated spectra and corresponding baselines, ML model training settings and parameter configurations, examples of OP-optimized results, the local minimum effect in OP-airPLS and convergence analysis, common optimal region of log­(λ)–log­(τ) mappings for 12 spectral shapes, clustering of optimized parameters, comparison of the performances of different ML models, performance of ML-airPLS on compound baseline shapes, performance of ML-airPLS for noisy spectra, performance of ML-airPLS for experimental spectra (PDF)
