# Supplementary material for: Beyond Traditional airPLS: Improved Baseline Removal in SERS with Parameter-Focused Optimization and Prediction
Source: Anal Chem. 2025 Jul 26;97(30):16211–8. doi: 10.1021/acs.analchem.5c01253 (PMC12367207; doi:10.1021/acs.analchem.5c01253)
Supplement: Supplementary file 1 [file ac5c01253_si_001.pdf]

Supporting Information

Beyond Traditional airPLS: Improved Baseline Removal in SERS with Parameter-Focused Optimization and Prediction

Jiaheng Cui [a], Xianyan Chen [b], Yiping Zhao [c]*

[a] School of Electrical and Computer Engineering, College of Engineering, The University of Georgia, Athens, GA, USA 30602

[b] Department of Epidemiology & Biostatistics, College of Public Health, The University of Georgia, Athens, GA, USA 30602

[c] Department of Physics and Astronomy, The University of Georgia, Athens, GA, USA 30602

* Corresponding Author: E-mail: zhaoy@uga.edu

Table of Contents

S1. Additional information of DP-airPLS. S3
S2. Issues with DP-airPLS S6
S3. Steps of the OP-airPLS algorithm. S8
S4. Improvements to the original official Python code. S11
S5. Simulated spectra and corresponding baselines. S12
S6. ML model training settings and parameter configurations. S19
S7. Examples of OP-optimized results. S23
S8. The local minimum effect in OP-airPLS and convergence analysis. S24
S9. Common optimal region of $\log(\lambda) - \log(\tau)$ mappings for 12 spectral shapes. S29
S10. Clustering of optimized parameters. S31
S11. Comparison of the performances of different ML models. S34
S12. Performance of ML-airPLS on compound spectral shapes. S38
S13. Performance of ML-airPLS for noisy spectra. S42
S14. Performance of ML-airPLS for experimental spectra. S47

S1. Additional information of DP-airPLS.

The airPLS algorithm [1] predicts baselines by iteratively optimizing a loss function, which balances fidelity to the observed spectrum and smoothness of the predicted baseline. The three key parameters governing the quality of baseline fitting are λ, p, and τ. The penalizing parameter λ is defined in the loss function Q, which can be expressed as:

$$Q(\boldsymbol{b}; \boldsymbol{s}, \boldsymbol{W}, \boldsymbol{D}, \lambda) = (\boldsymbol{s} - \boldsymbol{b})^T \boldsymbol{W} (\boldsymbol{s} - \boldsymbol{b}) + \lambda \|\boldsymbol{D}^p \boldsymbol{b}\|_2^2 , \tag{S1}$$

where $\boldsymbol{s} = [s_1, s_2, \dots, s_m]^T$ is the original spectrum with m wavenumbers, $\boldsymbol{b} = [b_1, b_2, \dots, b_m]^T$ is the predicted baseline, $\boldsymbol{W}$ is an $m \times m$ diagonal matrix representing weights, $\boldsymbol{D}^p$ represents the finite-difference matrix with order p, and $\|\boldsymbol{x}\|_2 = \sqrt{\sum_{i=1}^{m} x_i^2}$ represents the L^2 norm of a vector $\boldsymbol{x}$ $= [x_1, x_2, \dots, x_m]^T$. The first term in **Equation S1** represents fidelity, ensuring $\boldsymbol{b}$ closely approximates the spectrum $\boldsymbol{s}$. The second term, weighted by λ, penalizes deviations from smoothness. Larger λ values yield relatively smoother baselines as more weight is given to the penalty term.

The finite-difference matrix $\boldsymbol{D}^p$ governs the order of smoothness enforced on $\boldsymbol{b}$ [2], it is an $(m - p) \times m$ matrix. Its (i, j)-th entry is defined by

$$\boldsymbol{D}_{i,j}^p = \begin{cases} 0 , & j = 1, 2, \dots, i - 1 \\ (-1)^{p-(j-i)} \dfrac{p!}{(j - i)!\,(p - j + i)!} , & j = i, i + 1, \dots, i + p \end{cases} \qquad i = 1, 2, \dots, m - p. \tag{S2}$$

As a result, the first-order difference matrix $\boldsymbol{D}^1$ (size: $(m - 1) \times m$) is written as:

$$\boldsymbol{D}^1 = \begin{pmatrix} -1 & 1 & & & \\ & -1 & 1 & & \\ & & \ddots & \ddots & \\ & & & -1 & 1 \end{pmatrix}, \tag{S3}$$

and the second-order difference matrix $\boldsymbol{D}^2$ (size: $(m - 2) \times m$) is expressed as:

$$\boldsymbol{D}^2 = \begin{pmatrix} 1 & -2 & 1 & & & \\ & 1 & -2 & 1 & & \\ & & \ddots & \ddots & \ddots & \\ & & & 1 & -2 & 1 \end{pmatrix}. \tag{S4}$$

Matrix $\boldsymbol{D}^1$ (default) can be considered as an operator that calculates the first-order differences of a spectrum. The minimization of **Equation S1** using $\boldsymbol{D}^1$ infers $\|\boldsymbol{D}^1 \boldsymbol{b}\|_2^2$ is governed by terms $b_{i+1} - b_i$. It encourages $b_{i+1} - b_i$ to be close to zero, meaning consecutive points of the baseline to be similar, i.e., $b_{i+1} \approx b_i$. However, minimizing **Equation S1** also requires consideration of balancing the first term, $(\boldsymbol{s} - \boldsymbol{b})^T \boldsymbol{W} (\boldsymbol{s} - \boldsymbol{b})$. As a result, not all differences $b_{i+1} - b_i$ are exactly

zero. Since the penalty does not consider any constraint in slope, discontinuities in the baseline can be acceptable. This explains why $p = 1$ produces a baseline that is piecewise linear with potentially sharp transitions at certain points.$p = 2$ results in $b_{i+2} - 2b_{i+1} + b_i$ in each element. The minimization of $||\boldsymbol{D}^2\boldsymbol{b}||_2^2$ encourages $b_{i+2} - 2b_{i+1} + b_i$ to be close to zero. This implies the slope of two consecutive points of the baseline are approximately equal, i.e., $b_{i+2} - b_{\mathrm{i}+1} \approx b_{\mathrm{i}+1} - b_i$. Such a condition guarantees that the first derivative of the predicted baseline exists and is smooth. Based on this, we will fix $p = 2$ in the following discussion to ensure that the calculated baseline is smooth.

The weight matrix $\boldsymbol{W}$ is an $m \times m$ diagonal matrix,

$$\boldsymbol{W} = \begin{pmatrix} w_1 & & & \\ & w_2 & & \\ & & \ddots & \\ & & & w_m \end{pmatrix}. \qquad (S5)$$

Initially, all weights $\{w_i\}_{i=1}^{m}$ are set to 1, implying equal contributions of intensities from every wavenumber. As the algorithm progresses, the weights are iteratively updated to reflect the influence of each wavenumber on the baseline correction process. This adjustment ensures that the minimization of the objective function Q depends on $\boldsymbol{W}$. Since Q is a convex function with respect to $\boldsymbol{b}$, there exists a unique minimizer $\boldsymbol{b}$ for given $\boldsymbol{s}, \lambda, \boldsymbol{W}$, and $\boldsymbol{D}$ based on $\frac{\partial Q}{\partial \boldsymbol{b}} = 0$,

$$\boldsymbol{b} = (\boldsymbol{W} + \lambda \boldsymbol{D}^T \boldsymbol{D})^{-1} \boldsymbol{W} \boldsymbol{s}. \qquad (S6)$$

The weights are updated after iteration to penalize overestimated regions, using the following formula:

$$w_i = \begin{cases} 0 \quad , & b_i \leq s_i \\ \exp\left(\dfrac{t * (s_i - b_i)}{||\boldsymbol{d}||_1}\right), & b_i > s_i \end{cases} \qquad i = 1, 2, \ldots, m\,, \qquad (S7)$$

where t is the iteration count, $\boldsymbol{d} = \min(0, \boldsymbol{s} - \boldsymbol{b})$ captures negative elements of the residual $\boldsymbol{s} - \boldsymbol{b}$, and $||\boldsymbol{x}||_1 = \sum_{i=1}^{m} |x_i|$ represents the L^1 norm of the vector $\boldsymbol{x}$. After updating $\boldsymbol{W}$, the algorithm checks if the iteration needs to be stopped by using the tolerance parameter τ: the calculation will terminate if $||\boldsymbol{d}||_1 < \tau * ||\boldsymbol{s}||_1$. So τ defines the convergence condition for airPLS. The significance of τ is to ensure the negative value of the predicted spectrum is within a tolerance defined by τ. However, the DP-airPLS implemented a fixed τ at 0.001 [1].

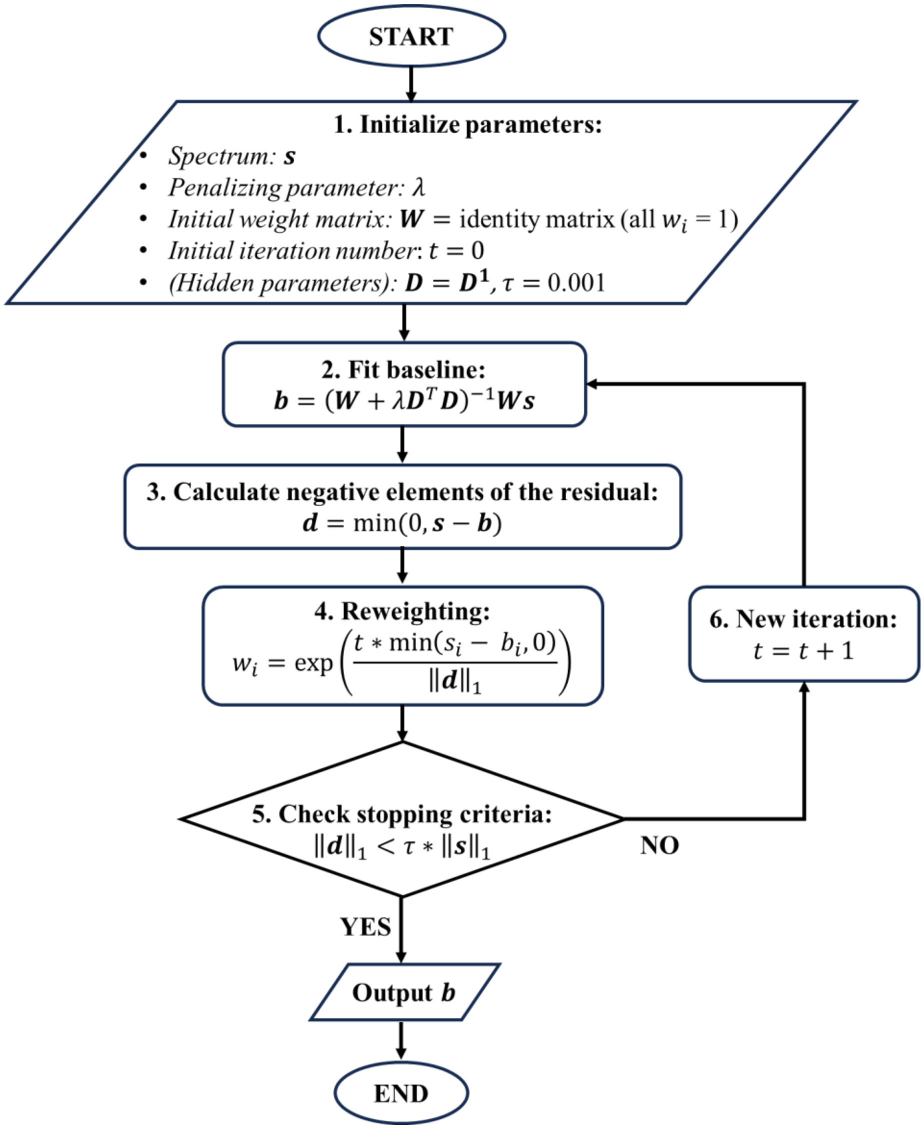

Figure S1. Flowchart of DP-airPLS.

S2. Issues with DP-airPLS.

Figure S2 provides detailed comparison analysis for an artificially generated spectrum (black dotted curve) with its true baseline (purple curve), the baseline estimated by DP-airPLS (orange curve) [1] as well as the baseline predicted by ModPoly 5 (green curve) [3]. It visualizes the three key problems with DP-airPLS baseline correction. The example spectrum represents a challenging case with broad overlapping peaks and complex baseline curvature that frequently occurs in SERS applications.

1) Non-smooth Baseline Artifacts: Baseline smoothness in airPLS depends on p, which represents the order of the finite-difference matrix $\boldsymbol{D}^p$ applied to the predicted baseline. However, p was not explicitly mentioned in the original airPLS paper, but is only used in the official code. This default p leads to a piecewise linear baseline. In contrast, the baseline predicted by ModPoly 5 is smooth at all wavenumbers, since it is restricted to a fifth-order polynomial.

2) Quantitative Performance Analysis: Comprehensive MAE analysis reveals region-dependent performance variations. The MAE for DP-airPLS (MAE_{DP}) is 10.6, while ModPoly 5 has an $MAE_{ModPoly}$ of 13.5. However, when analyzing specific spectral regions, DP-airPLS shows systematic failures in broad peak areas (321-550 cm^{-1}, 793-983 cm^{-1}, and 1400-1800 cm^{-1}) where baseline estimation becomes challenging due to peak-baseline ambiguity. Excluding the artificial trough introduced by ModPoly in featureless regions (> 1800 cm^{-1}), regional analysis shows MAE_{DP} increases to 15.3 while $MAE_{ModPoly}$ decreases to 5.54, confirming that DP-airPLS struggles specifically with broad peak interpretation.

3) Complex Spectral Region Limitations: The original airPLS paper only one spectrum of distinct peaks with two simple types of baseline [1], leaving real-world Raman or SERS spectral shapes largely untested. Real SERS spectra often exhibit broad, overlapping peaks that challenge the algorithm's ability to distinguish peaks from baseline curvature. DP-airPLS tends toward systematic under-estimation in these regions because the default parameters prioritize fidelity over smoothness, causing the baseline to partially follow peak contours rather than underlying baseline trends.

These systematic failures demonstrate that successful airPLS implementation requires careful parameter tuning beyond the original default values. The competing requirements for smoothness (addressing Problem 1), peak-baseline discrimination (addressing Problem 2), and

complex spectral handling (addressing Problem 3) necessitate simultaneous optimization of λ, τ, and p parameters rather than relying on fixed default values.

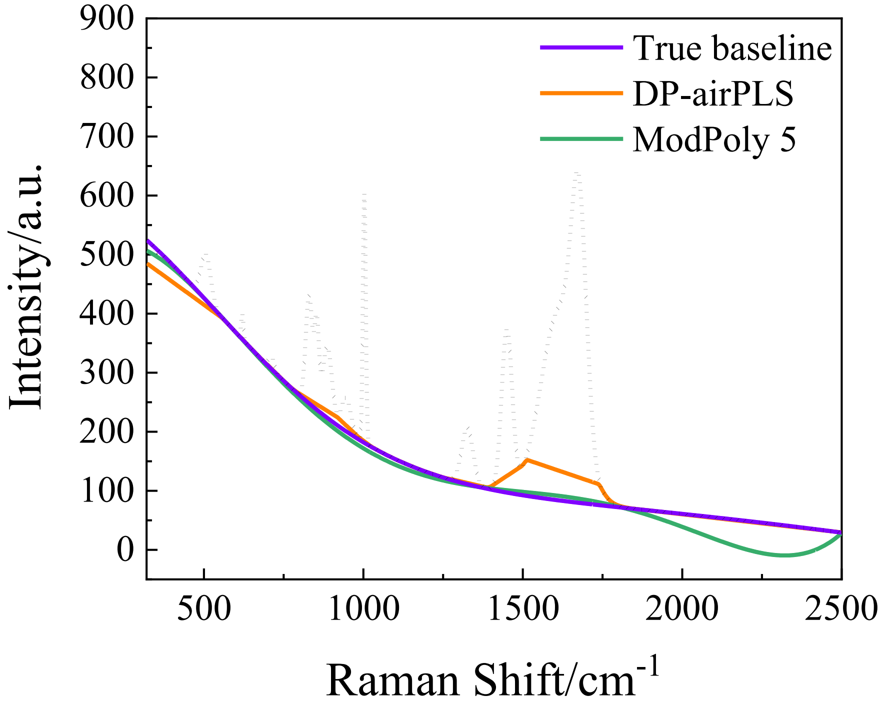

Figure S2. An artificially generated spectrum (black dotted curve) with its true baseline (purple curve) and predicted baselines obtained using two methods: DP-airPLS (orange curve) and ModPoly 5 (green curve).

We used the MAE to quantify the average absolute difference between $I_{pred}(\Delta\nu)$ and $I_{true}(\Delta\nu)$, over a total of m wavenumber points, calculated as:

$$MAE\left(I_{pred}(\Delta\nu), I_{true}(\Delta\nu)\right) = \frac{1}{m}\sum_{i=1}^{m}\left|I_{pred}(\Delta\nu_i) - I_{true}(\Delta\nu_i)\right| \qquad (S8)$$

S3. Steps of the OP-airPLS algorithm.

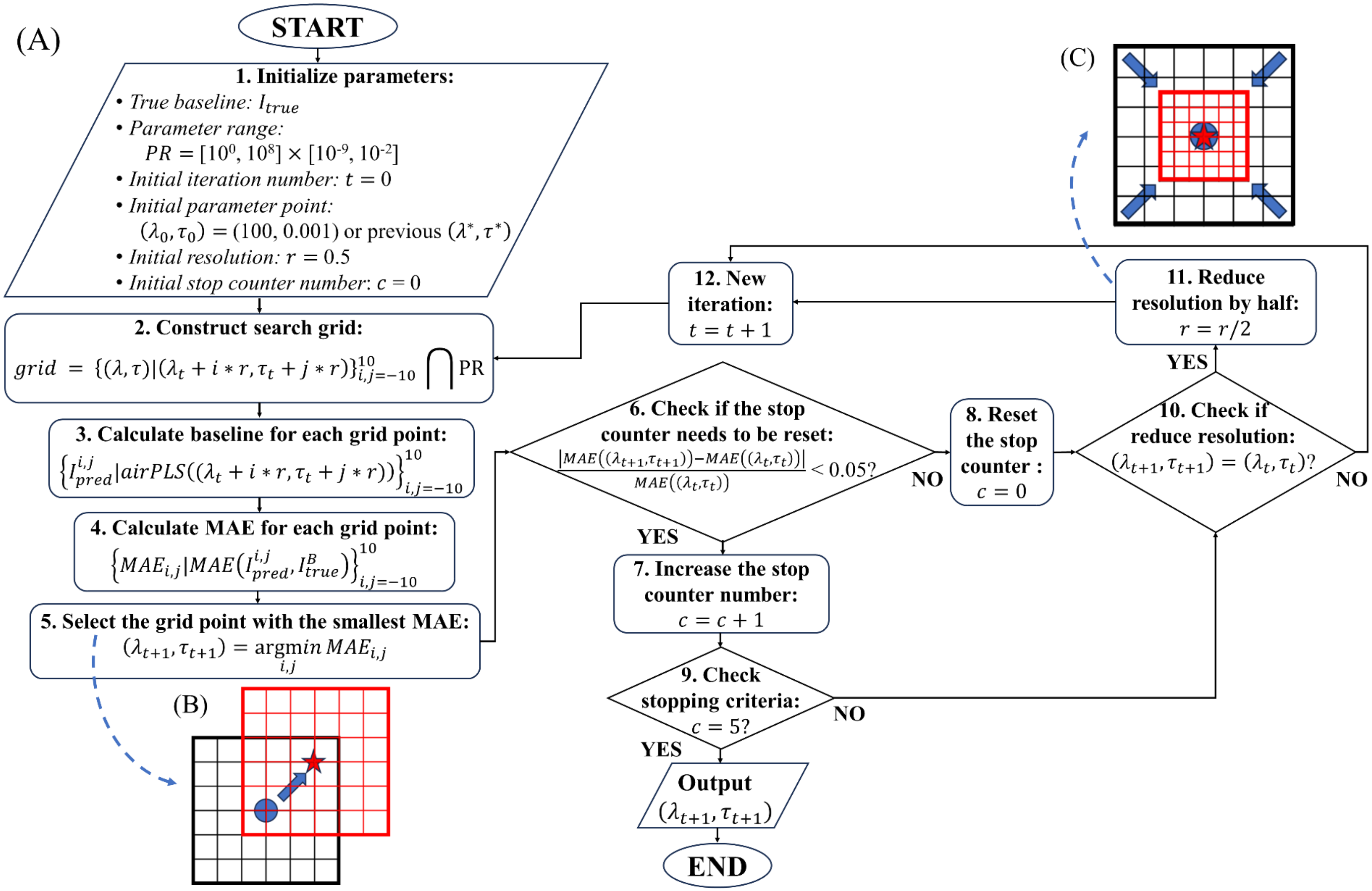

Figure S3. (A) Flowchart of the OP-airPLS algorithm. (B) Illustration of Step 5 (need to relocate the grid). (C) Illustration of Step 11 (need to reduce the resolution by half).

There are 12 key steps in the algorithm:

Step 1 (Initialize parameters): Suppose we are working on a spectrum with a true baseline I_{true}^{B}. The algorithm starts with iteration number $t = 0$. The initial parameter point is set to be (λ_0, τ_0). The parameter ranges are based on empirical observations and prior literature: $\log(\lambda) \in$ [0, 8], $\log(\tau) \in$ [-9, -2]. These ranges have been empirically determined to ensure that the tested parameter combinations are both computationally feasible and relevant for airPLS performance. Finally, we set the initial grid resolution parameter r to be 0.5, and the stop counter number c to be 0.

Step 2 (Construct search grid): In the t-th iteration, a 21 × 21 grid with a step size of r in both x- and y-direction, centered at $(\log(\lambda_t), \log(\tau_t))$ is constructed.

Step 3 (Calculate baseline for each grid point): For each grid point (λ, τ), the predicted baseline I_{pred}^{B} is computed using airPLS.

Step 4 (Calculate MAE for each grid point): For each grid point (λ, τ), the MAE between I_{pred}^{B} and the true baseline I_{true}^{B} is calculated.

Step 5 (Select the grid point with the smallest MAE): The grid point with the lowest MAE is selected as the new parameter center $(\lambda_{t+1}, \tau_{t+1})$.

Step 6 (Check if the stop counter needs to be reset): The algorithm checks if the relative change in MAE across consecutive iterations is less than 5%. This condition signifies that the optimization has potentially reached a stable solution within a local region. If this condition is satisfied, go to Step 7; otherwise, go to Step 8.

Step 7 (Increase the stop counter number): Let the stop counter number $c = c + 1$, then go to Step 9.

Step 8 (Reset the stop counter): The algorithm finds a new point that the MAE can be reduced by more than 5%. So, the current $(\lambda_{t+1}, \tau_{t+1})$ should not be considered as (λ^*, τ^*). Let c = 0, and directly go to Step 10.

Step 9 (Check stopping criteria): If c = 5, we have 5 stable iterations with the relative change in MAE is less than 5%. So $(\lambda_{t+1}, \tau_{t+1})$ is reported, and the algorithm is terminated. If not, go to Step 10.

Step 10 (Check if reduce resolution): If $(\lambda_{t+1}, \tau_{t+1})$ is the same as (λ_t, τ_t), then (λ_t, τ_t) is the global minimum given the current resolution. Then, go to Step 11; otherwise, directly go to Step 12.

Step 11 (Reduce resolution by half): The resolution r is halved ($r = r/2$). This refinement process ensures that the search focuses on a narrower region as the algorithm converges, thereby efficiently converging toward a local minimum.

Step 12 (New iteration): Let $t = t + 1$. Then, go back to Step 3, and repeat the whole process until the algorithm terminates in Step 9.

S4. Improvements to the original official Python code.

The proposed Python code introduced several critical improvements over the original DP-airPLS, enhancing its robustness, numerical stability, and usability:

1) Default value of p: The smoothing parameter p was fixed at 2 instead of 1.

2) Increased iteration limit: The maximum number of iterations was increased from 15 to 1000, ensuring stable calculations, particularly for low τ values. The highest iteration count observed in our calculations was 776.

3) Overflow prevention: To prevent overflow errors in **Equation S7**, the exponential term $\frac{t*(s_i - b_i)}{\|\boldsymbol{d}\|_1}$ was capped at 709, which was calculated by the command '*round(np.log(np.finfo(float).max))*'. This safeguard is crucial, as overflow errors frequently occur in the original code when $\tau < 10^{-7}$. The original implementation does not account for large exponential values, making this a significant improvement.

4) Exception handling: All possible exceptions of Python code during calculations are caught, logged, and thrown out, ensuring users are informed of any issues encountered during execution.

5) Fallback for invalid MAE values: If the MAE calculation fails for a given parameter set, the value is assigned as '*np.inf*,' effectively eliminating the grid point as a candidate for the optimal solution. Since '*np.inf*' is always larger than any real number, it ensures invalid points are never selected.

S5. Simulated spectra and corresponding baselines.

Let M be the total number of Gaussian peaks in the spectrum. To generate the i-th peak, we used the following formula:

$$Peak_i\ (x; A_i, \mu_i, \sigma_i) = A_i exp\left(-\frac{(x-\mu_i)^2}{2\sigma_i^2}\right), \qquad i = 1, 2, \ldots, M, \qquad (S9)$$

where x, A_i, μ_i, σ_i represent the Raman shift, the amplitude, the center, and the standard deviation of the peak. Please note the "width" or the full width at half maximum (FWHM) of the peak is $FWHM = 2\sqrt{2ln2}\sigma$. To formally define peak shapes B, C, and D, we used the following criteria:

Let $\mu_{\mathrm{i}}, \sigma_{\mathrm{i}}$ and $\mu_{i+1}, \sigma_{\mathrm{i+1}}$ be the centers and standard deviations of two consecutive Gaussian peaks. Then, the term "peaks overlapping" is then defined as $\mu_{\mathrm{i}} + 2 \cdot \sigma_i \geq \mu_{\mathrm{i+1}} - 2 \cdot \sigma_{i+1}$. The distinct peak shape is defined as no pair of consecutive peaks satisfying the overlap condition. The convoluted peak shape is defined as at least one pair of consecutive peaks satisfying the overlap condition, but no three consecutive peaks overlap. The broad peak shape is defined as three or more consecutive peaks satisfying the overlap condition.

The exact generation procedure for the three peak shapes is as follows:

1) Broad peak shape (B): Frequently encountered in biological samples such as bacteria [4] or viruses [5-9], these peaks are less defined and spread over a wider spectral region. Our broad peak shape is based on the average virus spectrum of COV229E (at 25,000 PFU/mL). We selected 12 representative peaks out of 35 peaks calculated by SciPy's *'find_peaks'* function. The comparison between the synthetic and original spectra is shown in **Figure S4A**.

2) Convoluted peak shape (C): Found in spectra of fluorescent dyes like 1,2-bis(4-pyridyl) ethylene (BPE) [10], these spectra feature overlapping peaks, leading to more complex profiles. We analyzed the average spectrum of BPE (50 μM) using the *'find_peaks'* function, which identified 17 Gaussian peaks. From these, we selected 7 major peaks in the region of 1015-1646 cm^{-1}. To ensure representation in 400-1015 cm^{-1}, we also manually added three distinct peaks at $\Delta\nu$ = 461, 620, and 800 cm^{-1}. The comparison between the synthetic spectrum and the original BPE spectrum is shown in **Figure S4B**.

3) Distinct peak shape (D): Characterized by sharp, well-separated peaks, these spectra are commonly observed in systems dominated by linker molecules such as 4-mercaptophenol (MPh). This peak shape was derived from the convoluted peak shape in **Figure S4A** by removing $\Delta\nu =$ 1254 cm^{-1} and 1610 cm^{-1}, and shifting the peak at $\Delta\nu$ = 1527 cm^{-1} to 1426 cm^{-1} to ensure no

overlaps. The comparison between the convoluted peak and distinct peak spectra is shown in **Figure S4C**. All the precise parameters for generating Gaussian peaks of the three peak shapes are enumerated in **Table S1**. Each spectrum was generated by placing peaks according to these parameters, with non-peak regions set to zero.

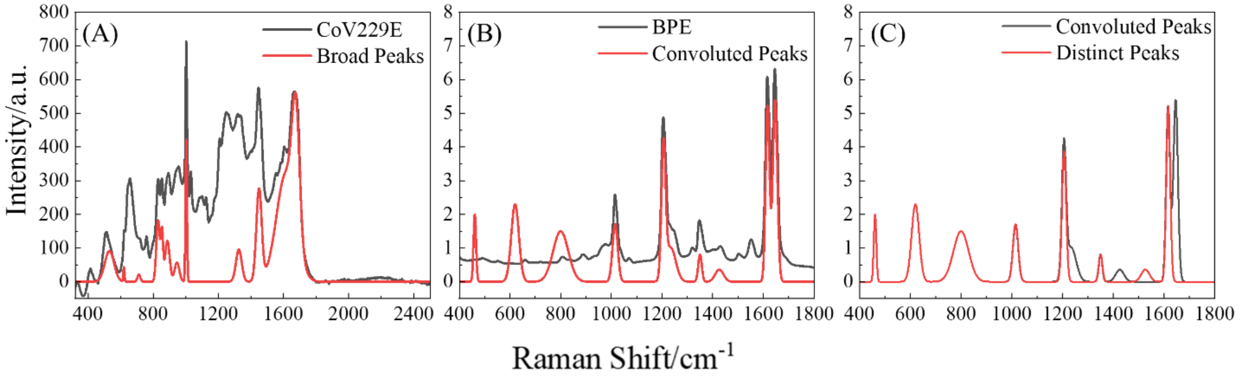

Figure S4. Definition of three peak shapes: (A) Fitted broad peak shape spectrum (red curve) vs average spectra of CoV229E (25,000 PFU/mL) (black curve). (B) Fitted convoluted peak shape spectrum (red curve) vs average spectra of BPE (50 μM) (black curve). (C) Fitted distinct peak shape spectrum (red curve) vs fitted convoluted peak shape spectrum (black curve).

Table S1. A summary of the peak parameters to generate the simulated spectra for 3 peak shapes, where I_B, I_C, I_D correspond to spectra of broad peak shape, convoluted peak shape, and distinct peak shape.

**Peak**	**1**	**2**	**3**	**4**	**5**	**6**	**7**	**8**	**9**	**10**	**11**	**12**
A_{I_B}	91	42	21	182	140	121	56	422	95	270	319	369
μ_{I_B}	531	620	712	830	854	887	946	1003	1326	1450	1615	1676
σ_{I_B}	28.34	3.66	9.01	10.70	6.86	13.94	14.72	4.52	17.83	16.37	58.55	23.26
A_{I_C}	2	2.3	1.5	1.71	3.87	1.02	0.81	0.36	5.2	5.39		
μ_{I_C}	461	620	800	1015	1206	1233	1350	1426	1617	1646		
σ_{I_C}	5	15	30	10.43	8.15	20.20	7.04	16.45	7.75	9.02		
A_{I_D}	2	2.3	1.5	1.71	3.87	0.81	0.36	5.2				
μ_{I_D}	461	620	800	1015	1206	1350	1527	1617				
σ_{I_D}	5	15	30	10.43	8.15	7.04	16.45	7.75				

The origin and mathematical formulations for each baseline type are as follows:

1) Modified exponential baseline (E): Used in simulations to model backgrounds that rapidly decrease from high to low intensities [11-15]. Physically, such baselines often arise from

fluorescence effects that diminish with increasing Raman shift, as seen in Ref. [8]. The math formula is: $a_1 e^{-b_1 x^2} + c_1 x^2 + d_1$.

2) Gaussian baseline (G): Frequently employed to model fluorescence backgrounds [11, 12, 15], where fluorescence intensity often exhibits a bell-shaped intensity distribution in certain chemical and biological systems. The math formula is: $Ae^{-\frac{(x-\mu 1)^2}{2\sigma^2}}$.

3) 5th-order polynomial baseline (P): A widely used choice in Raman and SERS data preprocessing [16-19], effectively capturing smooth, nonlinear background variations caused by scattering or instrumental artifacts. The math formula is: $a_2x^5 + b_2x^4 + c_2x^3 + d_2x^2 + ex + f$.

4) Sigmoidal baseline (S): Also used for fluorescence background modeling [12, 14, 17], these baselines exhibit an "S-shaped" intensity curve. The math formula is: $\frac{a_3}{1+e^{-b_3(x-\mu_2)}}$.

Here, x represents the Raman shift. To introduce variability, each baseline parameter ϕ in these formulas was perturbed using a Gaussian random distribution $\tilde{\phi} \sim N(\phi_0, (VC \cdot \phi_0)^2)$, i.e., the mean is ϕ_0, and the standard deviation is proportional to ϕ_0, scaled by a variation coefficient VC. **Table S2** summarizes all values for ϕ_0 and VC used for four baseline shapes.

Table S2. A summary of center values ϕ_0 and variation coefficient VC for all parameters ϕ for each of the 4 baseline shapes.

Baseline shape	ϕ	ϕ_0 (B)	ϕ_0 (C/D)	VC (B/C/D)
**E**	a_1	500	15	0.3
	b_1	1.6×10^{-6}	1×10^{-5}	0.3
	c_1	-2.7×10^{-6}	-1×10^{-7}	0.15
	d_1	100	2	0.05
**G**	A	1000	4	0.15
	μ_1	1500	1100	0.25
	σ	400	400	0.25
**P**	a_2	-6.25×10^{-14}	-6.76×10^{-15}	0.0005
	b_2	7.08×10^{-10}	4.90×10^{-11}	0.0005
	c_2	-2.65×10^{-6}	-1.30×10^{-7}	0.001
	d_2	4.22×10^{-3}	1.54×10^{-4}	0.002
	e	-3.18	-8.26×10^{-2}	0.005
	f	1414.81	20.61	0.05
**S**	a_3	1000	5.5	0.4
	b_3	0.005	0.008	0.4
	μ_2	1500	1100	0.1

To ensure sufficient spectral diversity, we systematically generated parameter combinations for each baseline type. For polynomial baselines (6 parameters), creating five variants per parameter resulted in 15,625 possible combinations. For exponential baselines (4 parameters), the same approach yielded 625 possible combinations. For Gaussian and sigmoidal baselines (3 parameters each), we conducted five independent sampling iterations, generating 125 spectra per iteration, leading to a total of 625 spectra per type. From these generated pools — 15,625 spectra for polynomial baselines and 625 spectra each for exponential, Gaussian, and sigmoidal baselines— we randomly selected 500 baselines per type to be combined with the three peak shapes, forming the final spectral dataset. All spectra were adjusted to ensure non-negative intensity values, and the complete set of generated spectra, along with their corresponding baselines, is presented in **Figure S5**.

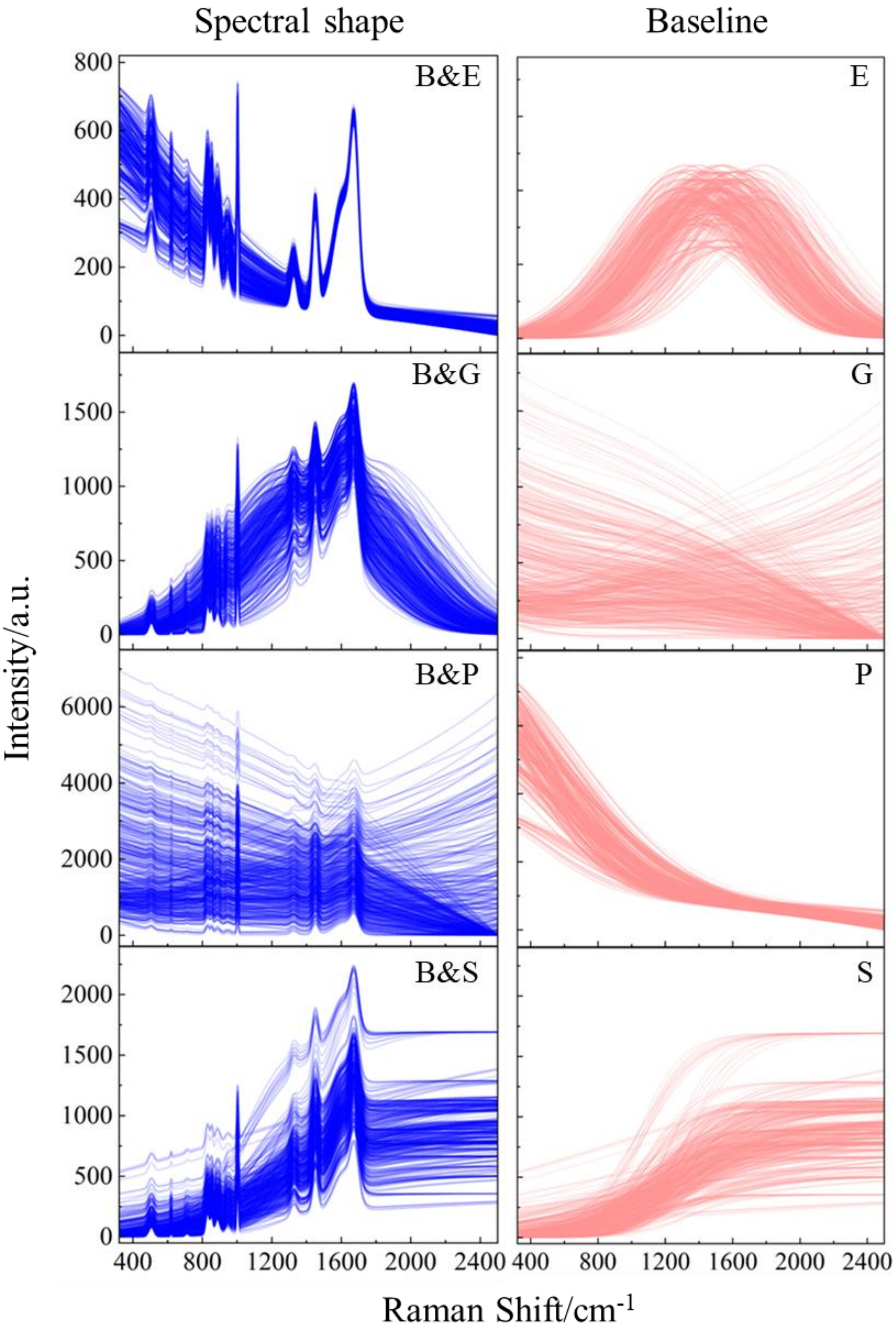
Spectral shape
Baseline
B&E
E
B&G
G
B&P
P
B&S
S
Intensity/a.u.
Raman Shift/cm-1
800
600
400
200
0
1500
1000
500
0
6000
4000
2000
0
2000
1500
1000
500
0
400
800
1200
1600
2000
2400
400
800
1200
1600
2000
2400

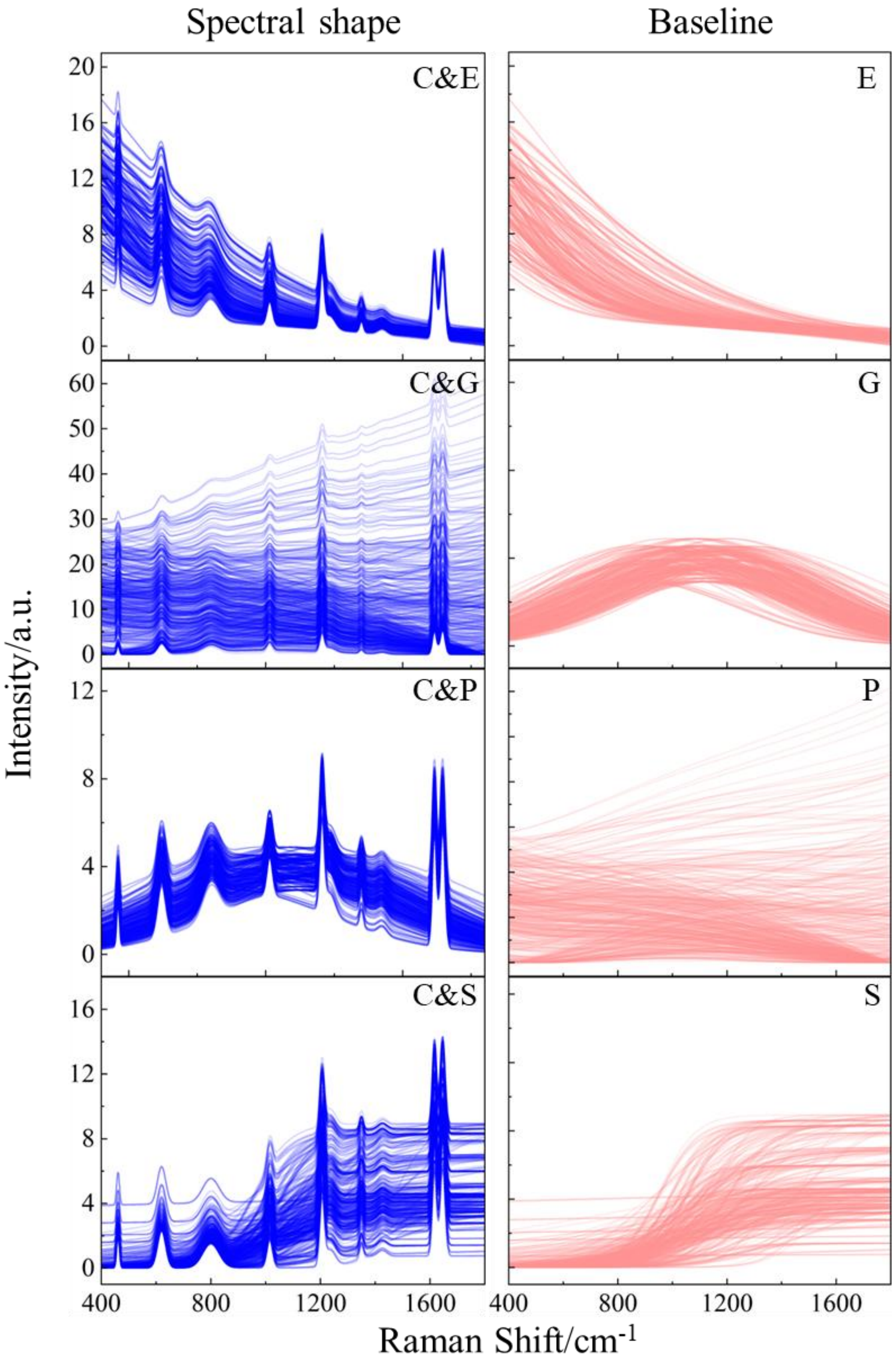

Spectral shape
Baseline
C&E
E
C&G
G
C&P
P
C&S
S
Intensity/a.u.
Raman Shift/cm-1
20
16
12
8
4
0
60
50
40
30
20
10
0
12
8
4
0
16
12
8
4
0
400
800
1200
1600
400
800
1200
1600

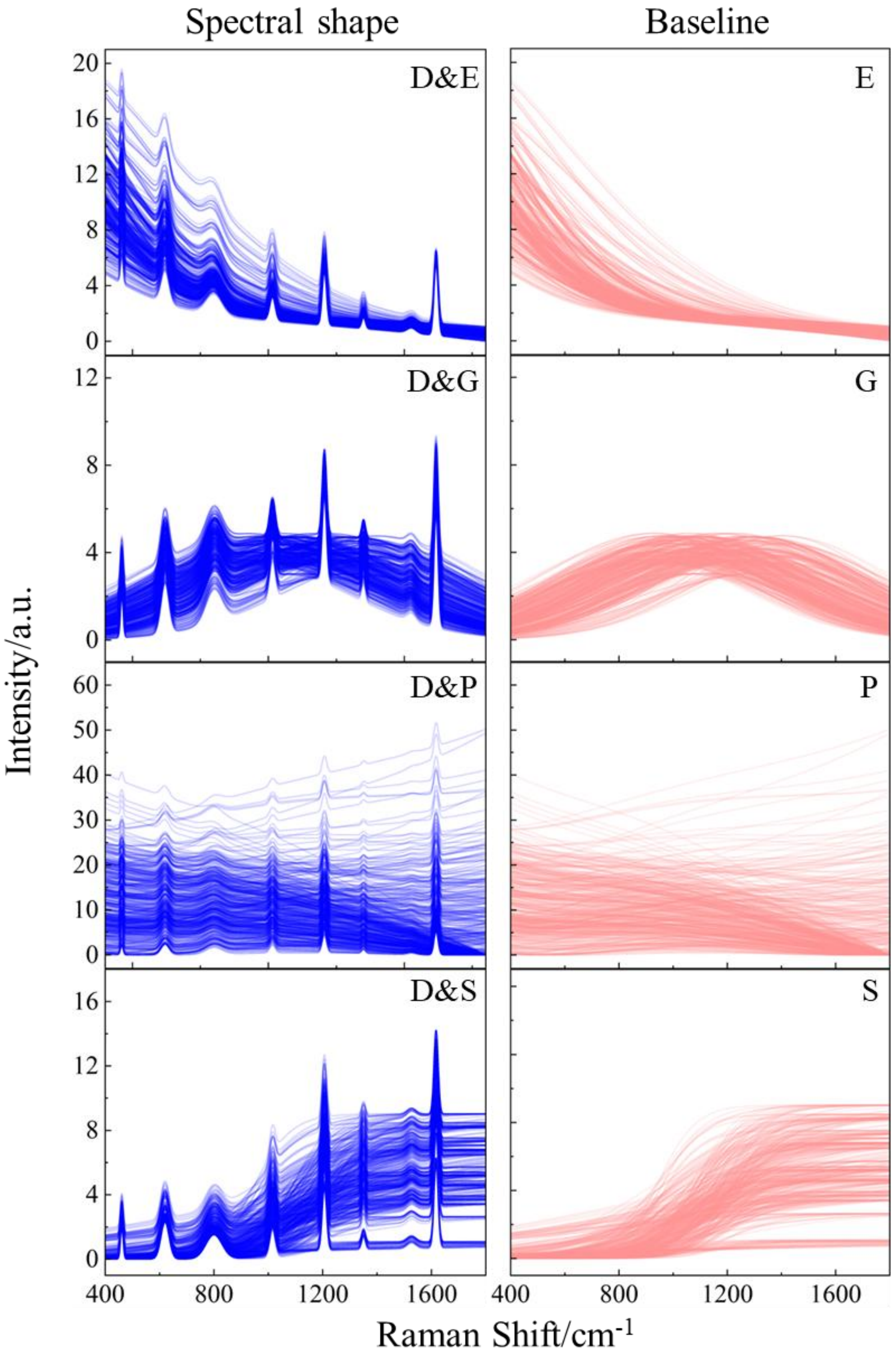

Figure S5. 500 generated spectra (Left) vs 500 corresponding baselines (Right) for 12 spectral shapes.

S6. ML model training settings and parameter configurations.

We evaluated multiple ML models for predicting (λ^*, τ^*) values, including Random Forest (RF) [20], Extreme Gradient Boosting (XGBoost) [21], Long Short-Term Memory (LSTM) [22], Deep Neural Network (DNN) [23], Convolutional Neural Network (CNN). For each algorithm type, we developed variants with and without principal component analysis (PCA) preprocessing. Additionally, each algorithm was implemented with multiple hyperparameter settings, resulting in 6 distinct configurations per algorithm, as detailed in **Table S3**. In total, we trained and evaluated 60 different training settings.

For the PCA-based models, we performed PCA on the training spectra to extract transformation coefficients for the first 10 principal components (PCs), which were then used to transform all training, validation, and testing spectra into their corresponding PC1 - PC10 values. The selection of 10 PCs was based on variance analysis, as this configuration captured 99.9999% of the total spectral variance (see **Table S4** for the cumulative variance for each PC). Notably, the first two PCs alone accounted for over 96% of the variance, highlighting the potential for significant dimensionality reduction while preserving essential spectral information. Each PCA-based ML model was trained using these 10 PCs as input features, with the corresponding (λ^*, τ^*) values from the training set as targets. The validation set was used for parameter tuning, and the final model performance was evaluated on the testing set.

Since λ and τ have different numerical scales, to establish a reasonable loss function, we applied logarithmic transformation followed by min-max normalization on these parameters before training. For each spectrum, the optimal parameters (λ^*, τ^*) were first converted to logarithmic scale $(\log \lambda^*, \log \tau^*)$. Then, these values were normalized to the range [0, 1] using min-max normalization:

$$\begin{cases} [\log \lambda_i^*]_N = \dfrac{\log \lambda_i^* - \min\limits_i \{\log \lambda_i^*\}_{i=1}^n}{\max\limits_i \{\log \lambda_i^*\}_{i=1}^n - \min\limits_i \{\log \lambda_i^*\}_{i=1}^n} \\ [\log \tau_i^*]_N = \dfrac{\log \tau_i^* - \min\limits_i \{\log \tau_i^*\}_{i=1}^n}{\max\limits_i \{\log \tau_i^*\}_{i=1}^n - \min\limits_i \{\log \tau_i^*\}_{i=1}^n} \end{cases}, i = 1, 2, \dots, 6000, \qquad (S10)$$

where the superscript N denotes normalized values. The ML models are trained to predict these normalized logarithmic parameters, denoted as $([\log \lambda_{i,pred}]_N, [\log \tau_{i,pred}]_N)$. During training, the loss function minimized the MAE between predicted and optimal parameters in the normalized space:

$$Loss = \frac{1}{n}\sum_{i=1}^{n}\left(\left|[\log \lambda_i^*]_N - \left[\log \lambda_{i,pred}\right]_N\right| + \left|[\log \tau_i^*]_N - \left[\log \tau_{i,pred}\right]_N\right|\right). \qquad (S11)$$

After obtaining predicted values, we transformed them back to their original scales. These final transformed parameters ($\lambda_{i,pred}, \tau_{i,pred}$) were then used as inputs to the airPLS algorithm for baseline removal. This transformation protocol ensures stable model training while maintaining the appropriate parameter ranges required by airPLS.

Table S3. A summary of the configurations for each ML model used in this study.

Model	Parameter	Config 1	Config 2	Config 3	Config 4	Config 5	Config 6
**RF**	Number of Trees	100	1000	5000	100	1000	5000
	Max Depth	None			20		
**XGBoost**	Number of Trees	100	1000	100	1000	100	1000
	Max Depth	6					
	Learning Rate	0.1		0.05		0.01	
**DNN**	Number of Layers	3	4	6	3	4	6
	Neurons per Layer	512, 1024, 512	512, 1024, 512, 128	512, 1024, 2048, 1024, 512, 128	512, 1024, 512	512, 1024, 512, 128	512, 1024, 2048, 1024, 512, 128
	Dropout Rate	0.3					
	Optimizer and Learning Rate	Adam, 0.001			Adam, 0.01		
	Batch Size	64					
	Epochs	500					
**LSTM**	Number of Layers	2	3	5	2	3	5
	Number of Neurons per Layer	64, 64	64, 128, 64	64, 128, 256, 128, 64	64, 64	64, 128, 64	64, 128, 256, 128, 64
	Activation Function	tanh					
	Dropout Rate	0.3					
	Optimizer and Learning Rate	Adam, 0.001			Adam, 0.01		
	Batch Size	64					
	Epochs	500					
**CNN**	Number of Layers	5	10	ResNet-50 [24]	5	10	ResNet-50
	Activation Function	ReLU					
	Dropout Rate	0.3			0.5		
	Kernel Size × Number of Filters	3×3×64, 3×3×64, 3×3×128, 3×3×256, 3×3×512	3×3×64, 3×3×64, 3×3×128, 3×3×256, 3×3×512, 3×3×512, 3×3×256, 3×3×128, 3×3×64, 3×3×64	Refer to Ref. [24]	3×3×64, 3×3×64, 3×3×128, 3×3×256, 3×3×512	3×3×64, 3×3×64, 3×3×128, 3×3×256, 3×3×512, 3×3×512, 3×3×256, 3×3×128, 3×3×64, 3×3×64	Refer to Ref. [24]
	Dropout Rate	0.3					
	Optimizer and Learning Rate	Adam, 0.001			Adam, 0.0001		
	Batch Size	64					
	Epochs	1000					

Table S4. A summary of the explained variance and cumulative variance for each PC in the PCA of the training spectral set.

PC	PC Explained Variance (%)	Cumulative Variance (%)
1	77.36	77.36
2	19.00	96.36
3	3.01	99.38
4	0.39	99.77
5	0.20	99.96
6	2.54×10^{-2}	99.99
7	7.91×10^{-3}	99.997
8	2.37×10^{-3}	99.9994
9	2.92×10^{-4}	99.9997
10	2.41×10^{-4}	99.9999
11	6.17×10^{-5}	99.99996
12	2.14×10^{-5}	99.99998
13	1.05×10^{-5}	99.99999
14	6.00×10^{-6}	> 99.99999
15	1.20×10^{-6}	> 99.99999

S7. Examples of OP-optimized results.

Figure S6B compares the baselines obtained by DP-airPLS and OP-airPLS. As expected, the DP-airPLS baseline is non-smooth, especially in the wavenumber regions between 320-500 cm^{-1} and 1350-1800 cm^{-1}. These irregularities are further highlighted in the first derivative of the predicted baseline (blue curve in **Figure S6C**), where multiple jumps occur. In contrast, the OP-airPLS baseline (red curve in **Figure S6B**) is smooth and continuous, as also confirmed by its first derivative (red curve in **Figure S6C**), with no abrupt changes. This smoothness validates the optimization algorithm's ability to achieve both accuracy and smoothness in baseline fitting.

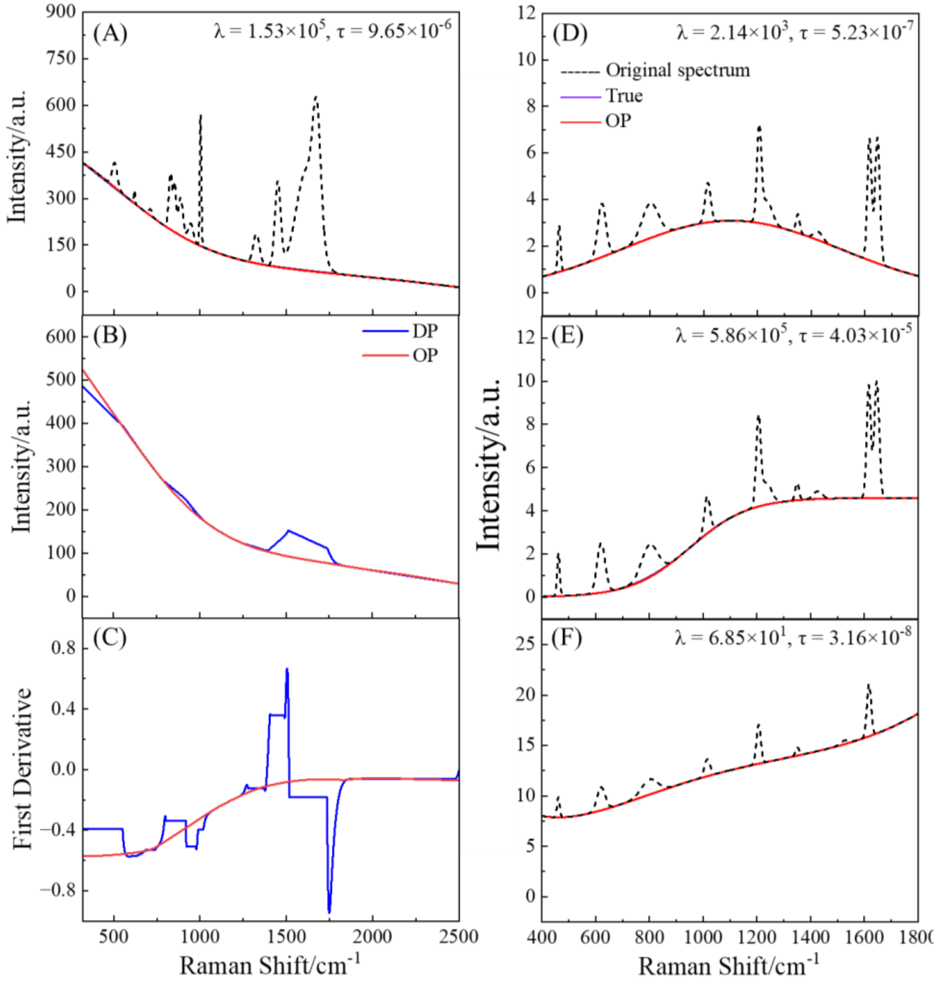

Figure S6. Comparison of the true baseline (purple curve) and the OP-airPLS estimated baseline (red curve): (A) A B&E spectrum, with a comparison of (B) baselines estimated by DP-airPLS (blue curve) and OP-airPLS (red curve) and (C) their corresponding first-order derivatives. Additionally, comparisons of the true baseline (purple curve) and OP-airPLS estimated baseline (red curve) are shown for a representative (D) C&G, (E) C&S, and (F) D&P spectrum.

S8. The local minimum effect in OP-airPLS and convergence analysis.

The nature of OP-airPLS guarantees that it can only converge to a local minimum, i.e., if different starting points (λ_0, τ_0) are chosen, the minimization of MAE may converge to different (λ^*, τ^*) points. **Figure S7A** shows a MAE map (50×50 mesh) of a B&S spectrum in the $\log(\lambda) - \log(\tau)$ plane, where $\log(\lambda) \in$ [0, 8] and $\log(\tau) \in$ [-9, -2]. The MAE is not a continuous function of $(\log(\lambda), \log(\tau))$, and three distinct regions, divided by the dashed lines in **Figure S7A**, can be identified:

Region I: Upper-left corner, above the black dotted line ($\log(\tau) > 0.893\log(\lambda) - 8$). The MAE values in this region are relatively high, ranging from 4.67 to 56.2.

Region II: Lower-right corner, below the red dotted line ($\log(\tau) < 0.815\log(\lambda) - 10$). This region has very large MAE values, with 95% of the MAE values > 93.7.

Region III: Valley region, between the black and red dotted lines. This region generally exhibits low MAE values, with 80% of the MAE values between 0.420 and 20.4. This is the region where most (λ^*, τ^*) are located.

To illustrate the local minimum effect, **Figure S7A** shows the convergence paths of 6 (λ_0, τ_0) points for a B&S spectrum: three initial points (Points #1 (0.5, -2.5), #4 (0.5, -5.5), and #5 (4, -8.5) converge to the white star ((1.09, -8.69), **Figure S7B**), while three others (Points #2 (2, -3), #3 (7.5, -2.5), and #6 (7.5, -8.5) converge to the red star ((4.18, -6.16), **Figure S7B'**). Both converged MAE_{OP} values, 0.422 and 0.861, represent a significant improvement (PI = 99.0% and 97.9%) compared to MAE_{DP} (40.7). Although the two local minima differ slightly, their proximity in terms of performance suggests that the algorithm produces acceptable results with negligible differences in different local minima. **Figure S7C** summarizes the number of iterations required for convergence across all 6 initial points. The optimization process reliably finds a minimum within five iterations, with an average convergence iteration number of 3 ± 1. The results demonstrate that the algorithm exhibits rapid and stable convergence, even in the presence of non-smoothness in the MAE surface.

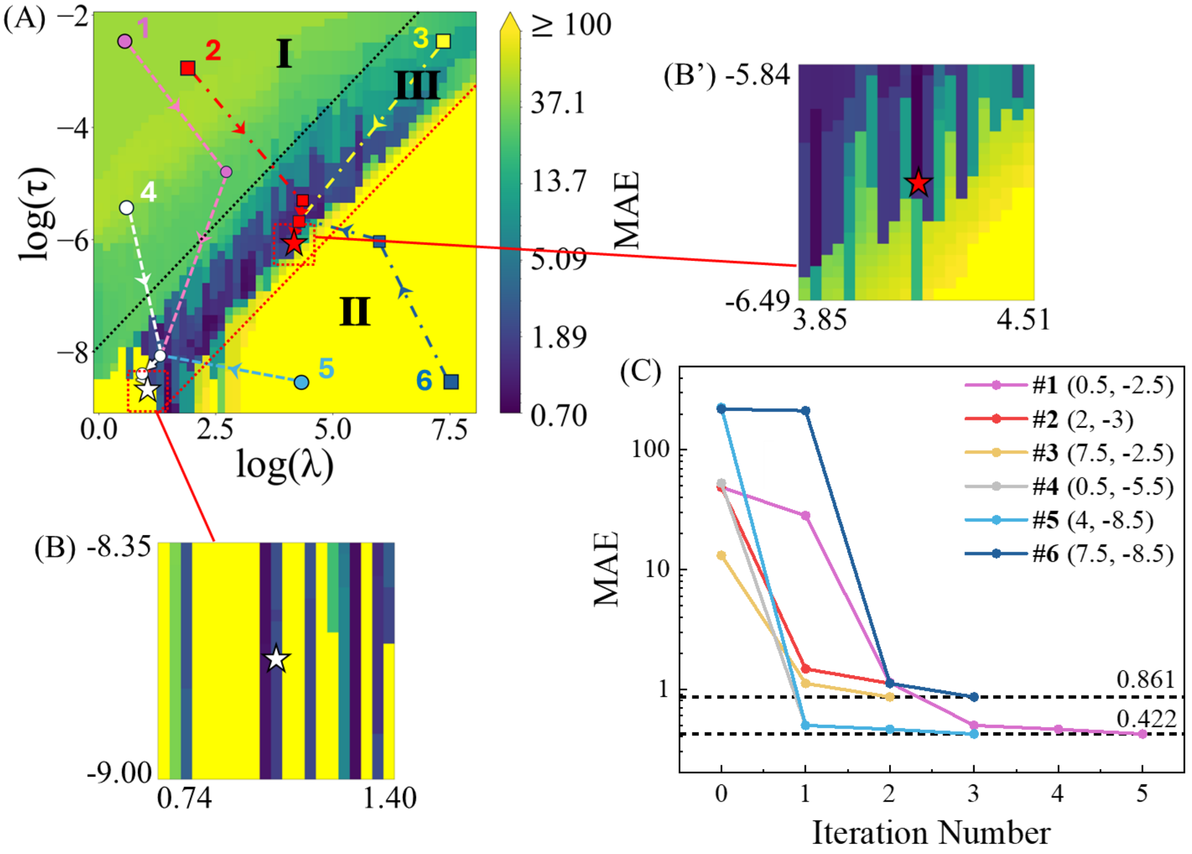

Figure S7. Convergence and local minima of OP-airPLS: (A) Six initial (λ_0, τ_0) points (circles & squares) are shown on a representative MAE map in the $\log(\lambda) - \log(\tau)$ plane for a B&S spectrum. The dot-dashed arrows indicate the convergence paths of each starting point, with 3 points (squares) converging to the red star and the other three (circles) converging to the white star. (B) and (B') show zoomed-in MAE maps for the two converged points, representing local minima. (C) The change in MAE as a function of iteration number during convergence for the six starting points.

To further illustrate the general conclusions for local minima, we select two spectra from each spectral shape (the spectra with the lowest and highest MAE_{DP} in each spectral shape). For each spectrum, 10 different (λ_0, τ_0) points are selected to study the convergence behavior: 9 of them are randomly selected from $\log(\lambda) \in [0.5, 7.5]$ and $\log(\tau) \in [-8.5, -2.5]$, and the 10th point is fixed at (2, -3), i.e., the parameters for DP-airPLS. **Figure S8** shows the MAE maps of one representative spectrum for each of the 12 spectral shapes. Similar to **Figure S7A**, all MAE maps display three distinct regions: the upper-left corner, the lower-right corner, and the diagonal valley

region. Importantly, all local minima are located within the diagonal valley region, as indicated in **Figure S8**. The optimal (λ^*, τ^*), MAE_{OP}, and PIs of each convergence point for each spectrum are summarized in **Table S5**. Most spectra exhibit two local minima, though some have one or three. For most cases, the MAE_{OP} values across different local minima are of the same order of magnitude. Compared to MAE_{DP}, PI is always larger than 90%. However, 6 out of 24 cases (indicated in red in **Table S5**) show significantly different MAE_{OP} values at different minima. These cases are outliers, and all have starting points located in the lower-right corner of the parameter space. In these instances, convergence is restricted to a local minimum in the lower-right corner, resulting in suboptimal performance. Based on this observation, we recommend avoiding initial points in the lower-right corner region. In fact, the best suggested initial values should be in Region III, the valley region.

The average number of convergence points for the 24 spectra is 1.6 ± 0.7, indicating that most spectra have one or two local minima. Although the algorithm is not guaranteed to converge to a single global minimum, the number of convergence points is small, and the performance at each convergence point is consistent. Excluding the outliers, the PIs for peak shapes B, C, and D are 93 ± 8 %, 96 ± 4 %, and 97 ± 2 %, respectively, suggesting that more complex peak shapes result in slightly lower PI values, which is expected. Nonetheless, the average PI for all peak shapes exceeds 90%, demonstrating the effectiveness of the optimization algorithm across diverse conditions. Furthermore, the small standard deviation in PI values indicates that the algorithm achieves similar performance regardless of convergence points. Finally, the average number of iterations required for convergence is 4 ± 2, which is comparable to the results in **Figure S7**. This consistency confirms that the optimization algorithm is both fast and stable.

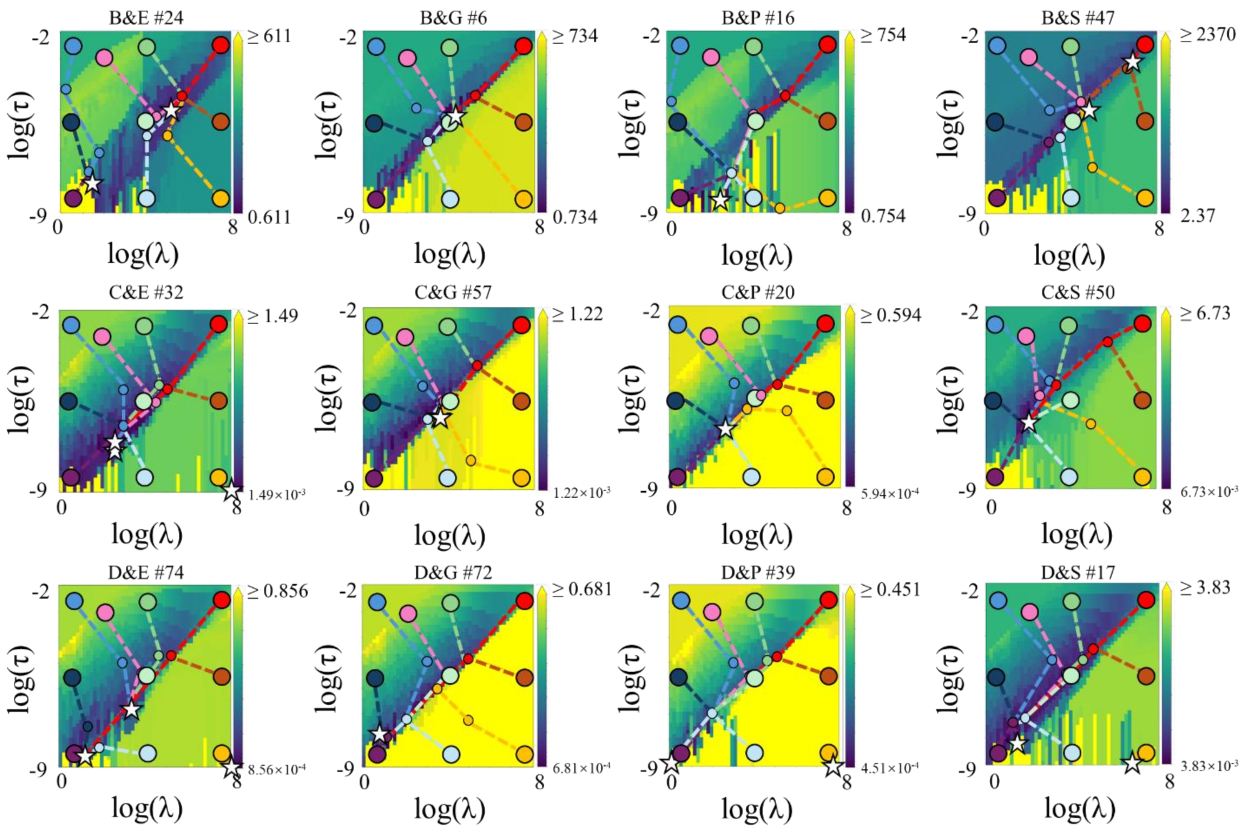

Figure S8. Local minima convergence mapping for one representative spectrum for each of the 12 spectral shapes. The "#" in the title of each figure indicates the number of the spectrum selected from the 500 spectra. Similar to **Figure S7A**, the visualized convergence results (λ^*, τ^*) (indicated by stars) for the 10 starting points are shown.

Table S5. A summary table of optimization results for two representative spectra from each spectral shape (with smallest and largest MAE_{DP}). For each spectrum, the table lists $(\log(\lambda^*), \log(\tau^*))$, MAE_{OP}, PI_{OP}, and the number of starting points converging to this local minimum. Values corresponding to outlier cases are highlighted in red.

Peak shape	Baseline shape	MAE case	MAE_{DP}	log (λ^*)	log (τ^*)	MAE_{OP}	PI_{OP}	# Converging points
B	E	Max	13.9	1.3	-7.77	0.372	97.3	3
				5.16	-5.03	1.04	92.5	7
		Min	9.08	5.19	-5	1.06	88.3	10
	G	Max	6.74	4.22	-5.44	0.625	90.7	10
		Min	25.9	4.12	-5.25	0.702	97.3	10
	P	Max	11.5	2.44	-8.56	0.417	96.4	10
		Min	48.9	2	-8	0.477	99.0	9
				4.22	-5.88	0.797	98.4	1
	S	Max	13.6	4.72	-4.75	1.60	88.3	9
				6.92	-3.07	3.94	71.1	1
		Min	40.7	1.09	-8.69	0.422	99.0	5
				4.18	-6.16	0.861	98.0	5
	**Avg**	-	-	-	-	-	**93 ± 8**	-
C	E	Max	0.113	2.62	-7.12	2.00×10^{-3}	98.2	8
				2.58	-7.28	2.00×10^{-3}	98.2	1
				8	-9	0.189	-67.6	1
		Min	0.0398	3.45	-6.08	1.99×10^{-3}	97.5	9
				6.5	-9	0.361	-806	1
	G	Max	0.0174	3.62	-6	7.29×10^{-4}	95.8	10
		Min	0.0302	3.41	-6.16	1.21×10^{-3}	96.0	7
				3.45	-6.14	1.21×10^{-3}	96.0	3
	P	Max	0.265	2.61	-6.73	5.94×10^{-4}	99.8	10
		Min	0.0252	2.88	-7.38	4.80×10^{-4}	98.1	10
	S	Max	0.0315	1.92	-6.36	5.00×10^{-3}	84.1	10
		Min	0.153	0.938	-8.31	4.15×10^{-3}	97.4	10
	**Avg (w/ outlier)**	-	-	-	-	-	**0 ± 300**	-
	**Avg (w/o outlier)**	-	-	-	-	-	**96 ± 4**	-
D	E	Max	0.0918	1.06	-8.62	8.02×10^{-4}	99.1	9
				8	-9	0.351	-282	1
		Min	0.0560	1	-8.59	7.60×10^{-4}	96.6	7
				3.25	-6.5	2.00×10^{-3}	96.4	2
				8	-9	0.360	-543	1
	G	Max	0.0832	1.53	-9	7.88×10^{-4}	99.1	10
		Min	0.0170	0.812	-7.81	5.13×10^{-4}	97.0	10
	P	Max	0.0771	0.625	-8.38	4.78×10^{-4}	99.4	10
		Min	0.0227	0	-8.88	4.51×10^{-4}	98.0	9
				7.75	-9	0.547	-2310	1
	S	Max	0.183	1.34	-8.03	2.76×10^{-3}	98.4	9
				6.75	-9	0.124	32.2	1
		Min	0.0229	1.47	-7.56	1.13×10^{-3}	95.6	6
				5.58	-3.27	1.13×10^{-3}	95.1	4
	**Avg (w/ outlier)**	-	-	-	-	-	**-200 ± 600**	-
	**Avg (w/o outlier)**	-	-	-	-	-	**97 ± 2**	-

S9. Common optimal region of $\log(\lambda) - \log(\tau)$ mappings for 12 spectral shapes.

To analyze the consistency of (λ^*, τ^*) distributions for different spectral shapes, we defined an overall "common small MAE region" $R_{overall}$ using the following three steps:

Step 1: Two spectra were selected for each spectral shape: one with the largest MAE_{DP} and one with the smallest MAE_{DP}, as shown in **Table S5**. The parameter space ($\log(\lambda) \in$ [0, 8], $\log(\tau) \in$ [-9, -2]) was divided into a 50×50 grid evenly. Let the resolutions in the λ and τ directions be r_λ and r_τ, respectively. Then, for grid point (i, j), the corresponding parameter values (λ_i, τ_j) are:

$$\begin{cases} \lambda_i = exp(i \cdot r_\lambda) \\ \tau_j = exp(-9 + i \cdot r_\tau) \end{cases}, i, j = 0, 1, \ldots, 49. \qquad (S12)$$

We define the "small MAE region" (R_k) of the k-th spectrum as:

$$R_k = \left\{(i,j) \middle| MAE_k\big((\lambda_i, \tau_j)\big) \leq 10 * \min_{i,j} MAE_k \right\}, k = 1, 2, \ldots, 24, \qquad (S13)$$

where $MAE_k((\lambda_i, \tau_j))$is the MAE between the predicted baseline for the k-th spectrum using parameter (λ_i, τ_j) and the corresponding true baseline, $\min_{i,j} MAE_k$ indicates the minimum MAE achievable for the k-th spectrum. For the selected 12 representative spectra in **Figure S8**, the small MAE regions are shown in white in **Figure S9.**

Step 2: The indicator function for each R_k is defined as:

$$\mathbb{I}_{R_k}(i,j) = \begin{cases} 1, & (i,j) \in R_k \\ 0, & otherwise \end{cases} \qquad (S14)$$

Step 3: The common small MAE region for a given percentage threshold θ of the overall 24 spectra $R_{overall}(\theta)$ can be defined as:

$$R_{overall}(\theta) = \left\{(i,j) \middle| \sum_{k=1}^{24} \mathbb{I}_{R_k}(i,j) \geq \lceil 24 * \theta \rceil \right\}, \qquad (S15)$$

where "$\lceil 24 * \theta \rceil$" represents the ceiling operation of $24 * \theta$, i.e., rounded up to the nearest integer. The heatmap in **Figure 2A** shows the mapping of $R_{overall}(\theta)$ as the background, illustrating how the region evolves with varying θ. We selected a threshold $\theta = 70\%$ to define the common small MAE region, $R_{overall} \stackrel{\text{def}}{=} R_{overall}(0.7)$, which includes 73 out of the 2500 mesh points. This threshold provides a balance between capturing the key convergence points and excluding outliers. Points in $R_{overall}$ are colored red in **Figure 2A**. $R_{overall}$ also corresponds to the red ellipse in **Figure 2A** and highlights promising starting points for the majority of spectral shapes. It can be characterized using a linear equation, as described in **Equation 3**.

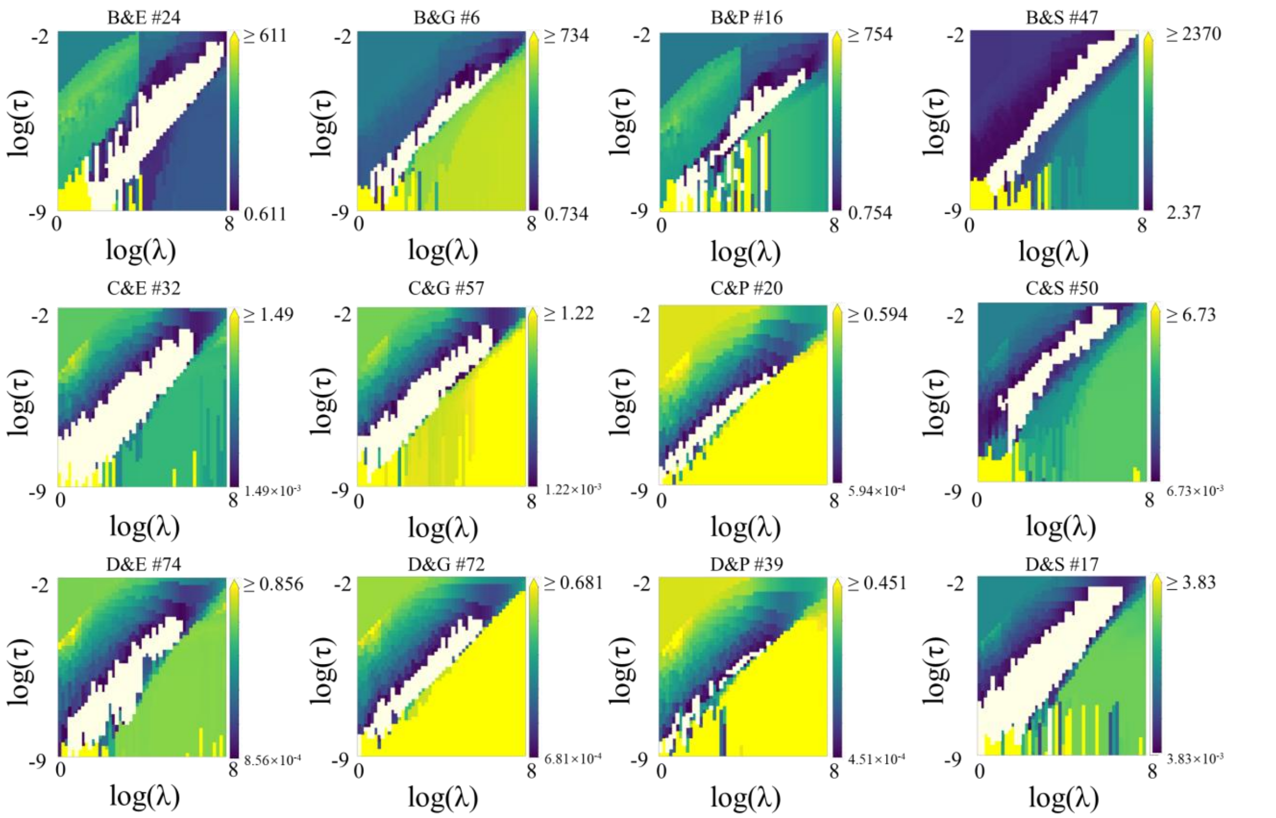

Figure S9. Distribution of “small MAE region” R_k (white areas) overlaid on MAE mappings for representative spectra of each spectral shape.

S10. Clustering of optimized parameters.

We observed that the obtained (λ^*, τ^*) points cluster closely together for each spectral shape, as shown in **Figure 2A**. Thus, a natural choice for the default parameters of each spectral shape could either be the logarithmic center (LOC, $(\lambda_{LOC}, \tau_{LOC})$) or the linear center (LIC, $(\lambda_{LIC}, \tau_{LIC})$).

For each spectral shape, let (λ_i^*, τ_i^*) be the optimized parameters for the i-th spectrum ($i = 1, 2, \dots, n$, and $n = 500$). Then, $(\lambda_{LOC}, \tau_{LOC})$ is calculated as,

$$\begin{cases} \log(\lambda_{LOC}) = \dfrac{1}{n}\displaystyle\sum_{i=1}^{n} \log(\lambda_i^*) \\ \log(\tau_{LOC}) = \dfrac{1}{n}\displaystyle\sum_{i=1}^{n} \log(\tau_i^*) \end{cases}. \quad (S16)$$

And $(\lambda_{LIC}, \tau_{LIC})$ is expressed as,

$$\begin{cases} \lambda_{LIC} = \dfrac{1}{n}\displaystyle\sum_{i=1}^{n} \lambda_i^* \\ \tau_{LIC} = \dfrac{1}{n}\displaystyle\sum_{i=1}^{n} \tau_i^* \end{cases}. \quad (S17)$$

The LOC (black stars) and LIC (purple stars) of two spectral shapes, B&E and B&P, are visualized as examples in **Figures S10A-C** and **S10A'-C'**, respectively. In **Figure S10A**, the distribution of the 500 optimized parameters (λ^*, τ^*) for B&E exhibits a tight cluster with its 95% confidence ellipse (the purple ellipse in **Figure 2A**) capable of covering nearly all of (λ^*, τ^*). It is evident that the LOC and the LIC are in close proximity to each other, both being situated near the center of the cluster. In contrast, the LOC and LIC for B&P (**Figure S10A'**) are well separated. In fact, LOC is situated centrally between two sub-clusters of (λ^*, τ^*), while LIC is situated in proximity to one of the sub-clusters. Therefore, neither the LOC nor the LIC can be considered representative of the overall (λ^*, τ^*) distribution.

Using both LOC and LIC as default (λ, τ) values, we conducted a systematic investigation of their influence on baseline fitting. **Figure S10B** shows the predicted and true spectra of a B&E spectrum (i.e., the spectrum in **Figures S2** and **S6**) using both LOC (red) and LIC (green), and **Figure S10C** shows the predicted and true baseline. In this case, the LOC and LIC positions are very close to each other ($(\log(\lambda_{LOC}), \log(\tau_{LOC})) = (5.11, -5.05)$, $(\log(\lambda_{LIC}), \log(\tau_{LIC})) = (5.19, -4.98)$), and they lead to similar results. Visually, all the extracted spectra overlap with the

true spectra, and the extracted baselines match very well with the true baseline. The corresponding MAE_{DP} = 10.6, MAE_{OP} = 0.746 (PI = 93.0%), MAE_{LOC} = 3.70 (PI = 62.9%), MAE_{LIC} = 4.92 (PI = 50.6%), respectively. Though MAE_{LOC} and MAE_{LIC} are smaller than MAE_{OP}, compared to MAE_{DP}, they cannot be considered significant improvements, but they could still be considered acceptable. In contrast, **Figures S10B'-C'** illustrate the challenges encountered in a B&G spectrum, where the predicted baseline and spectrum based on both LOC and LIC deviate significantly from the true baseline and spectrum. In particular, the Gaussian peak in the baseline appears to be underestimated for both LOC and LIC, resulting in elevated spectrum in 2000-2500 cm^{-1}. In this example, MAE_{DP} = 7.79, MAE_{OP} = 1.89 (PI = 77.0%), MAE_{LOC} = 13.33 (PI = -71.5%), MAE_{LIC} = 21.48 (PI = -176.4%). Since PI_{LOC} and PI_{LIC} are < -70%, both LOC and LIC's performance are unacceptable in this instance.

Figure 2B provides the summary of the MAE_{LOC} (purple bars) and MAE_{LIC} (yellow bars) for all 12 spectral shapes. For LOC, two spectral shapes — B&E and B&P — achieve a reduction in MAE of at least 60%, with average PI values of 60 ± 10% and 80 ± 20%, respectively. For LIC, three spectral shapes — B&E, B&P, and B&S — exhibit a reduction in MAE of at least 60%, with average PI values of 60 ± 10%, 80 ± 10%, and 60 ± 20%, respectively. For the remaining spectral shapes, PI values are 50% or less, with some average PI values even being negative. Given the instability or inadequacy (or both) of overall PI in most spectral shapes, we do not recommend using LOC or LIC as default parameters in general.

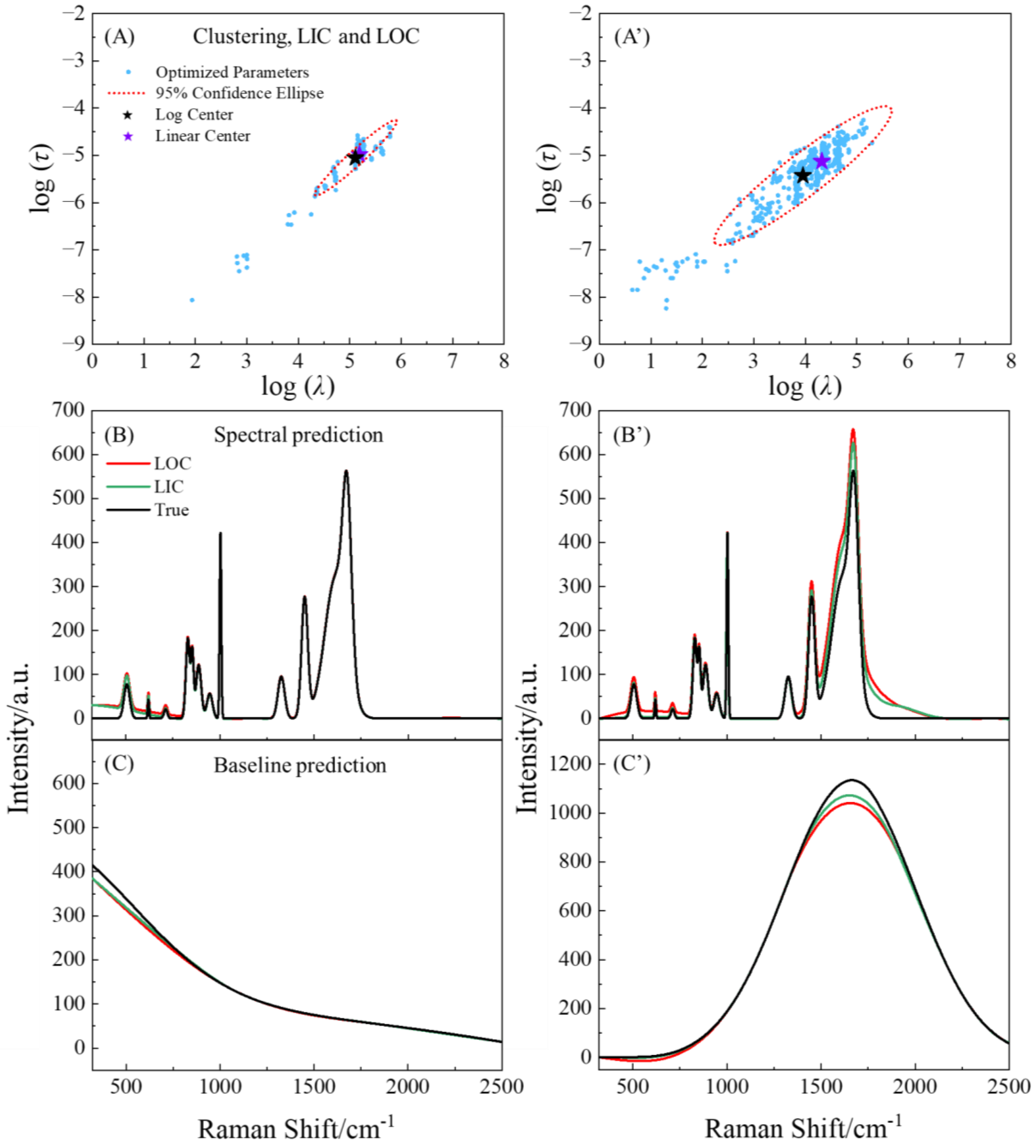

Figure S10. (A) The locations of the LOC (black star), LIC (purple star), and a 95% confidence ellipse (red dotted ellipse) for the (λ^*, τ^*) clustering (blue dots) of the 500 spectra for the (A) B&E and (A') B&G sepctral shapes. Predicted (B) spectra and (C) baselines for a representative B&E spectrum using LOC (red curve) and LIC (green curve) for airPLS, along with the true spectra and baselines (black curve). Predicted (B') spectra and (C') baselines for a B&P representative spectrum using LOC (red curve) and LIC (green curve) for airPLS, along with the true spectra and baselines (black curve). An obviously underestimated peak in both predicted baselines is shown.

S11. Comparison of the performances of different ML models.

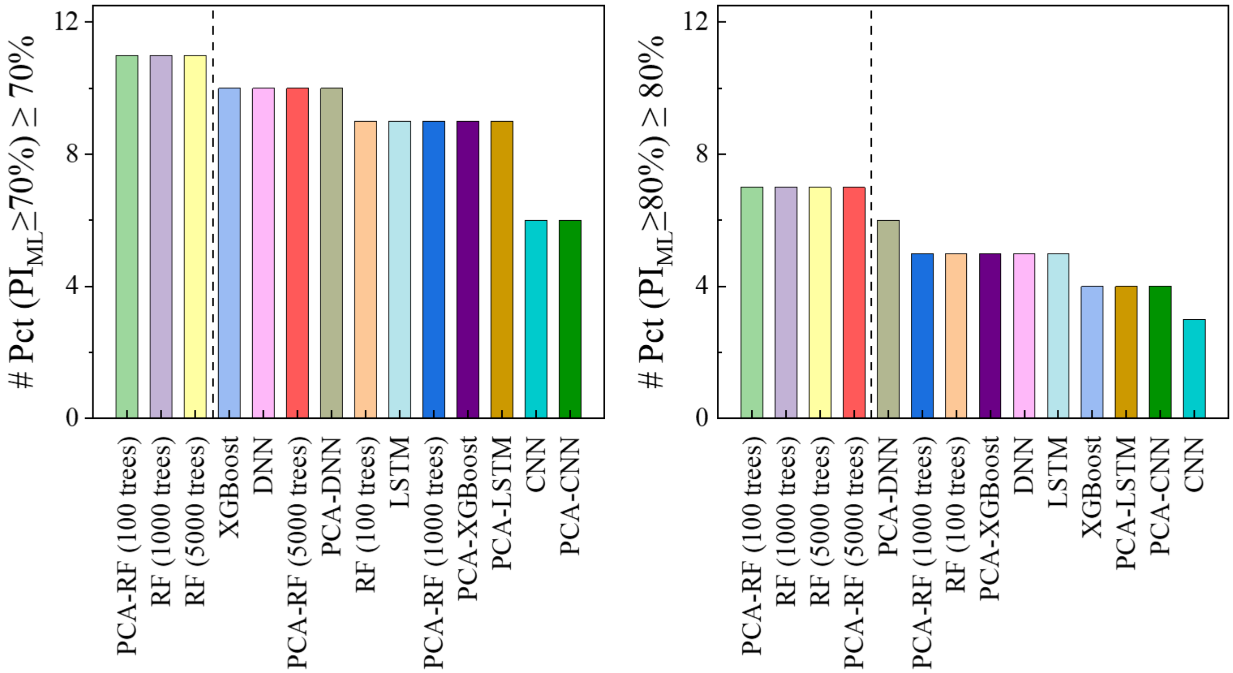

Figure S11. The number of spectral shapes with (A) $Pct\ (PI_{ML} \geq 70\%) \geq 70\%$ and (B) $Pct\ (PI_{ML} \geq 80\%) \geq 80\%$ for each ML model. For XGBoost, DNN, LSTM, and CNN, either with or without PCA, only the best candidates of each model are shown here.

Table S6. A summary of the Pct values at thresholds of $PI_{ML} \geq 70\%$ and $PI_{ML} \geq 80\%$ for all 12 spectral shapes for ML models PCA-RF, RF-1000, and RF-5000. The best performances for each spectral shape are highlighted in bold. The number of best-performed spectral shapes of each model is listed in the last row.

Metric	Pct ($PI_{ML} \geq 70\%$)			Pct ($PI_{ML} \geq 80\%$)		
**Model**	PCA-RF	RF-1000	RF-5000	PCA-RF	RF-1000	RF-5000
**Overall**	**84.7**	84.0	83.7	**79.5**	79.2	78.7
**B&E**	**100**	98	**100**	**100**	98	98
**B&G**	**84**	**84**	82	82	**84**	82
**B&P**	**92**	86	88	**90**	82	82
**B&S**	86	84	**90**	80	82	**86**
**C&E**	**98**	96	**98**	**96**	94	**96**
**C&G**	**74**	**74**	**74**	**70**	64	66
**C&P**	90	**94**	88	88	**94**	88
**C&S**	**86**	82	84	72	72	**74**
**D&E**	94	94	**96**	90	**92**	90
**D&G**	**68**	64	58	**64**	56	54
**D&P**	72	**76**	72	66	**68**	66
**D&S**	72	**76**	74	56	**64**	62
**Count**	**7**	5	5	**5**	**5**	3

Observations of predicted parameter positions by ML-airPLS for each spectral shape can be found in **Figure S12**. While the predicted positions (red) are within the cluster of real positions (blue) for each spectral shape, the corresponding MAE_{ML} may be large and become outliers. We defined outliers quantitatively based on the distribution of MAE_{ML} within each spectral shape. For typical predictions, MAE_{ML} was observed to be 1-2 orders of magnitude lower than MAE_{DP}, so does the median MAE_{ML} of each spectral shape. Based on this observation and visual inspection of prediction quality, we classified a prediction as an outlier if its MAE_{ML} exceeded 10 times the median MAE_{ML} of its corresponding spectral shape. This threshold effectively identified cases where ML predictions significantly deviated from the expected performance. For example, we examined three representative cases from the C&G spectral shape, which has a median MAE_{ML} of 1.20×10^{-3}. A clear outlier is the spectrum with the largest MAE_{ML} (1.41, approximately 1174.7 times the median), which yielded a PI of -4553.7%, demonstrating severe prediction failure. For a borderline case, we identified the spectrum with the smallest MAE_{ML} that just exceeds our threshold: MAE_{ML} = 1.36×10^{-2} (11.3 times the median) resulting in PI = 54.5%. We also examined

the spectrum with the largest MAE_{ML} that falls just below our threshold: MAE_{ML} = 6.70×10^{-3} (5.6 times the median) with PI = 76.9%, which maintains acceptable performance. These boundary cases validate our 10× median threshold as a reasonable balance for outlier detection. The number of outliers identified for each spectral shape is summarized in row 8 of **Table S7**.

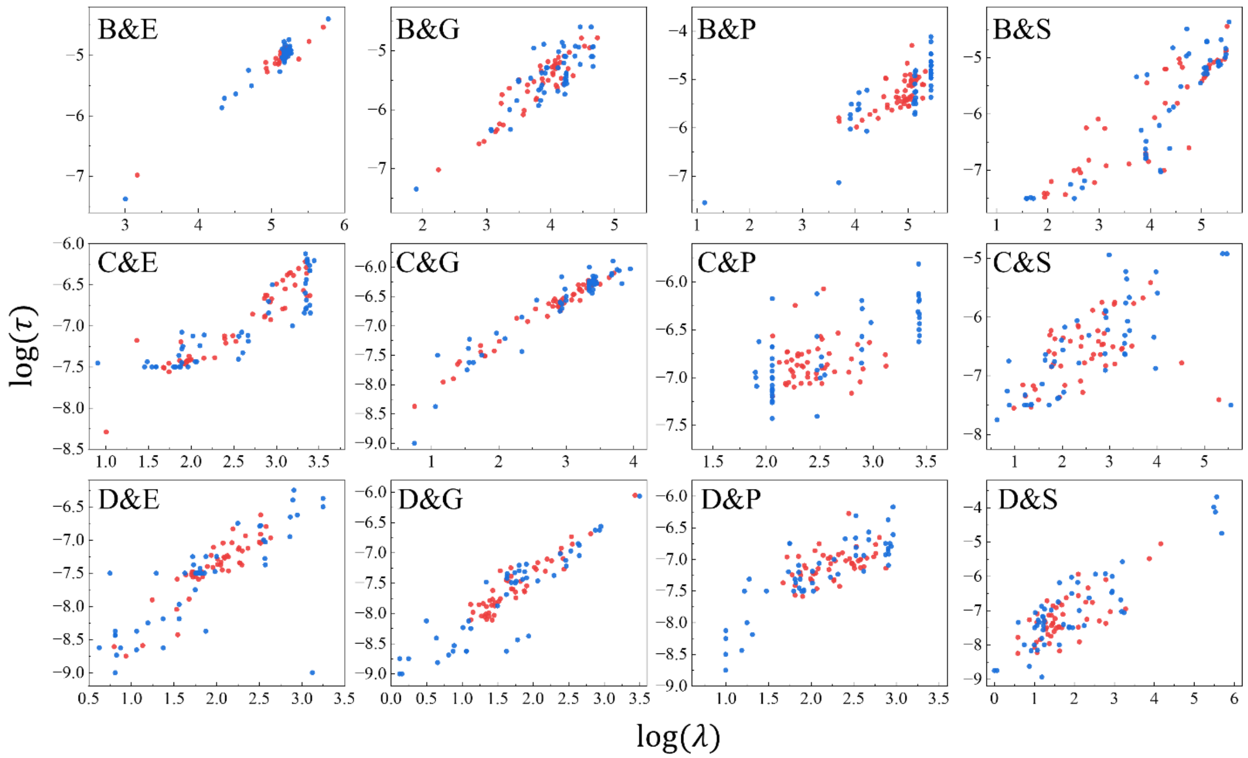

Figure S12. The distribution of predicted positions by ML $(\log(\lambda_{ML}), \log(\tau_{ML}))$ (red dots) vs $(\log(\lambda^*), \log(\tau^*))$ (blue dots) in $\log(\lambda) - \log(\tau)$ map for the testing spectra of all spectral shapes.

Table S7. A summary of cluster regions, average MAE and PI values, number of outliers in machine learning, and computation time per spectrum based on DP-airPLS (abbreviated as "DP"), OP-airPLS (abbreviated as "OP"), LOC, LIC, and ML-airPLS (abbreviated as "ML", refer to PCA-RF model) for all 12 spectral shapes. Poorly performed spectral shapes (only exist in LOC, LIC, and ML) are marked in red.

Metric		B&E	B&G	B&P	B&S	C&E	C&G	C&P	C&S	D&E	D&G	D&P	D&S	Avg
$(\log(\lambda^*), \log(\tau^*))$ region		[4, 6] × [−6, −4]	[3, 5] × [−6, −4]	[4, 6] × [−6.3, −4]	[2.5, 6] × [−6.5, −4]	[1.4, 3.5] × [−7.5, −6]	[0.5, 4] × [−8.5, −6.7]	[1.5, 3.5] × [-7.5, -5.5]	[0, 5.5] × [−7.5, −5]	[0, 4] × [−9, −6]	[0, 3] × [-9, -6]	[1, 3] × [-8.5, -6]	[0, 6] × [-9, -4]	-
MAE	DP	11 ± 2	15 ± 6	32 ± 9	26 ± 8	0.07 ± 0.02	0.026 ± 0.003	0.10 ± 0.07	0.06 ± 0.03	0.07 ± 0.01	0.03 ± 0.01	0.05 ± 0.02	0.06 ± 0.04	-
	OP	0.8 ± 0.1	0.7 ± 0.4	1.5 ± 0.3	0.8 ± 0.3	0.0014 ± 0.0005	0.0009 ± 0.0004	0.00055 ± 0.00005	0.003 ± 0.003	0.00 ± 0.02	0.0001 ± 0.0001	0.0005 ± 0.0002	0.004 ± 0.003	-
	ML (w/o outlier)	0.8 ± 0.2	1 ± 1	3 ± 2	2 ± 2	0.003 ± 0.003	0.002 ± 0.002	0.0013 ± 0.0006	0.004 ± 0.003	0.005 ± 0.004	0.003 ± 0.004	0.006 ± 0.008	0.01 ± 0.02	-
	LOC	5 ± 2	10 ± 10	6 ± 3	10 ± 20	0.06 ± 0.02	0.02 ± 0.01	0.03 ± 0.01	0.1 ± 0.1	0.07 ± 0.02	0.3 ± 0.2	0.03 ± 0.02	0.1 ± 0.1	-
	LIC	5 ± 1	10 ± 10	6 ± 4	10 ± 4	0.07 ± 0.02	0.019 ± 0.006	0.03 ± 0.01	0.04 ± 0.04	0.06 ± 0.02	0.02 ± 0.01	0.04 ± 0.02	0.05 ± 0.04	-
	ML (w/ outlier)	0.8 ± 0.2	6 ± 30	3 ± 2	10 ± 10	0.0 ± 0.2	0.1 ± 0.3	0.01 ± 0.03	0.01 ± 0.02	0.007 ± 0.010	0.01 ± 0.03	0.02 ± 0.02	0.02 ± 0.04	
	# ML outlier	0	5	0	6	2	11	7	7	2	7	9	4	5 ± 3
PI	OP	93.5 ± 0.9 %	95 ± 4 %	95 ± 3 %	96 ± 6 %	98.2 ± 0.6 %	97 ± 1 %	99.0 ± 0.5 %	94 ± 4 %	98.6 ± 0.5 %	95 ± 4 %	98.9 ± 0.5 %	93 ± 5 %	96 ± 2 %
	ML (w/o outlier)	93 ± 2 %	90 ± 10 %	90 ± 10 %	92 ± 9 %	96 ± 4 %	93 ± 7 %	98 ± 2 %	89 ± 6 %	92 ± 7 %	90 ± 10 %	90 ± 20 %	80 ± 30 %	90 ± 10 %
	LOC	60 ± 10 %	10 ± 60 %	80 ± 20 %	30 ± 70 %	20 ± 10 %	20 ± 40 %	50 ± 30 %	-200 ± 200 %	-20 ± 20 %	-1000 ± 1000 %	30 ± 30 %	-200 ± 200 %	-100 ± 100 %
	LIC	60 ± 10 %	30 ± 30 %	80 ± 10 %	60 ± 20 %	20 ± 10 %	30 ± 20 %	40 ± 30 %	10 ± 10 %	9 ± 5 %	-3 ± 7 %	20 ± 20 %	-0.0 ± 1.0 %	30 ± 20 %
	ML (w/ outlier)	93 ± 2 %	100 ± 100 %	90 ± 10 %	80 ± 50 %	100 ± 200 %	-300 ± 1000 %	80 ± 70 %	60 ± 90 %	90 ± 10 %	80 ± 40 %	60 ± 80 %	70 ± 50 %	100 ± 300 %
Calculation Time (s)	DP	0.015 ± 0.001	0.013 ± 0.002	0.016 ± 0.001	0.016 ± 0.002	0.013 ± 0.002	0.013 ± 0.002	0.011 ± 0.002	0.012 ± 0.002	0.012 ± 0.002	0.012 ± 0.001	0.011 ± 0.002	0.012 ± 0.002	0.013 ± 0.002
	OP	50 ± 20	80 ± 30	110 ± 90	70 ± 40	100 ± 80	70 ± 40	120 ± 90	90 ± 50	60 ± 50	40 ± 40	100 ± 100	50 ± 70	80 ± 70
	LOC	0.06 ± 0.01	0.057 ± 0.006	0.074 ± 0.009	0.071 ± 0.006	0.095 ± 0.002	0.085 ± 0.007	0.16 ± 0.02	0.12 ± 0.03	0.10 ± 0.09	0.11 ± 0.04	0.15 ± 0.05	0.13 ± 0.05	0.10 ± 0.05
	LIC	0.09 ± 0.02	0.05 ± 0.03	0.07 ± 0.06	0.05 ± 0.03	0.12 ± 0.05	0.13 ± 0.02	0.089 ± 0.007	0.10 ± 0.04	0.10 ± 0.02	0.06 ± 0.03	0.140 ± 0.006	0.07 ± 0.03	0.09 ± 0.04
	ML	0.037 ± 0.003	0.036 ± 0.002	0.037 ± 0.003	0.038 ± 0.004	0.036 ± 0.002	0.036 ± 0.003	0.037 ± 0.003	0.039 ± 0.002	0.037 ± 0.003	0.038 ± 0.004	0.041 ± 0.003	0.039 ± 0.003	0.038 ± 0.003

S12. Performance of ML-airPLS on compound spectral shapes.

Real-world spectra often exhibit combinations of the baseline and peak characteristics used in our training dataset. To evaluate ML-airPLS robustness on such complex scenarios, we constructed compound spectral shapes that systematically combine multiple baseline types and peak characteristics beyond the discrete categories used in model training.

1) Compound baseline construction: Our four individual baseline shapes exhibit three distinct spectral behaviors across the Raman shift range: exponential (E) and polynomial (P) both decrease from left to right, Gaussian (G) exhibits a bell-shaped profile with a central maximum, and sigmoidal (S) increases from left to right. Based on these distinct trends, we designed compound baseline combinations to represent realistic scenarios where multiple physical phenomena occur simultaneously. We created four compound baseline combinations by adding pairs of individual baselines (1:1 ratio) with different spectral behaviors: E+P (combining two decreasing trends), E+G (decreasing + bell-shaped), E+S (decreasing + increasing), and G+S (bell-shaped + increasing). We selected one representative spectrum from each baseline type and added these two individual baselines to create the compound baseline combinations.

2) Compound peak shape construction: Since distinct peaks are already covered in both broad and convoluted peak categories, we don't need to specifically add distinct peaks to the compound peak shape. To further increase spectral complexity, we constructed a compound peak shape (B+C) by adding the broad and convoluted peak shape spectra together, creating more complex spectral features than either individual peak type alone. The peak locations and parameters are detailed in **Table S8**.

3) Overall experimental design: We generated 12 compound spectra covering three combinations: (1) the original peak shape B with four different compound baselines, (2) the original peak shape C with four different compound baselines, (3) the compound peak shape (B+C) with all compound baseline types.

Table S8. A summary of the peak parameters to generate the simulated spectra for the compound peak shape B+C, where I_{B+C} correspond to the spectrum of the compound peak shape.

**Peak**	**1**	**2**	**3**	**4**	**5**	**6**	**7**	**8**	**9**	**10**	**11**	**12**	**13**	**14**
$A_{\mathrm{I_{B+C}}}$	78	205	205	182	140	122	56	422	95	270	319	369	305	305
$\mu_{\mathrm{I_{B+C}}}$	500	716	746	900	924	957	1016	1073	1326	1450	1615	1676	2016	2046
$\sigma_{\mathrm{I_{B+C}}}$	15.2	7.74	9.02	10.7	6.86	13.9	14.7	4.52	17.8	16.4	58.6	23.3	7.74	9.02

Since we constructed these compound spectra with known individual components, the true compound baselines were available for direct comparison. We used the PCA-RF model to predict (λ^*, τ^*) parameters, applied these parameters to airPLS for baseline estimation, and calculated PI by comparing the airPLS-estimated baselines with the known true compound baselines. This approach differs from our training data evaluation, where grid search optimization was used to determine optimal parameters.

Figure S13 visualizes representative results for each compound baseline type across different peak shapes. Beyond demonstrating successful parameter prediction, the figure reveals important qualitative differences between baseline methods. Across all 12 compound spectra, DP-airPLS exhibits its fundamental weakness of producing discontinuous, piecewise linear baselines. This discontinuity is particularly evident in broad peak regions and at wavenumbers around 400-550 cm^{-1} for E+P baseline combinations and in C&(E+S) scenarios. In contrast, ML-predicted baselines maintain smoothness throughout the spectral range, consistent with the $p = 2$ smoothness constraint. While ML-airPLS demonstrates superior smoothness, minor baseline overestimation occasionally occurs at isolated peaks, such as the peak at ~1000 cm^{-1} in convoluted peak spectra. However, these deviations remain within acceptable ranges and do not compromise overall baseline quality.

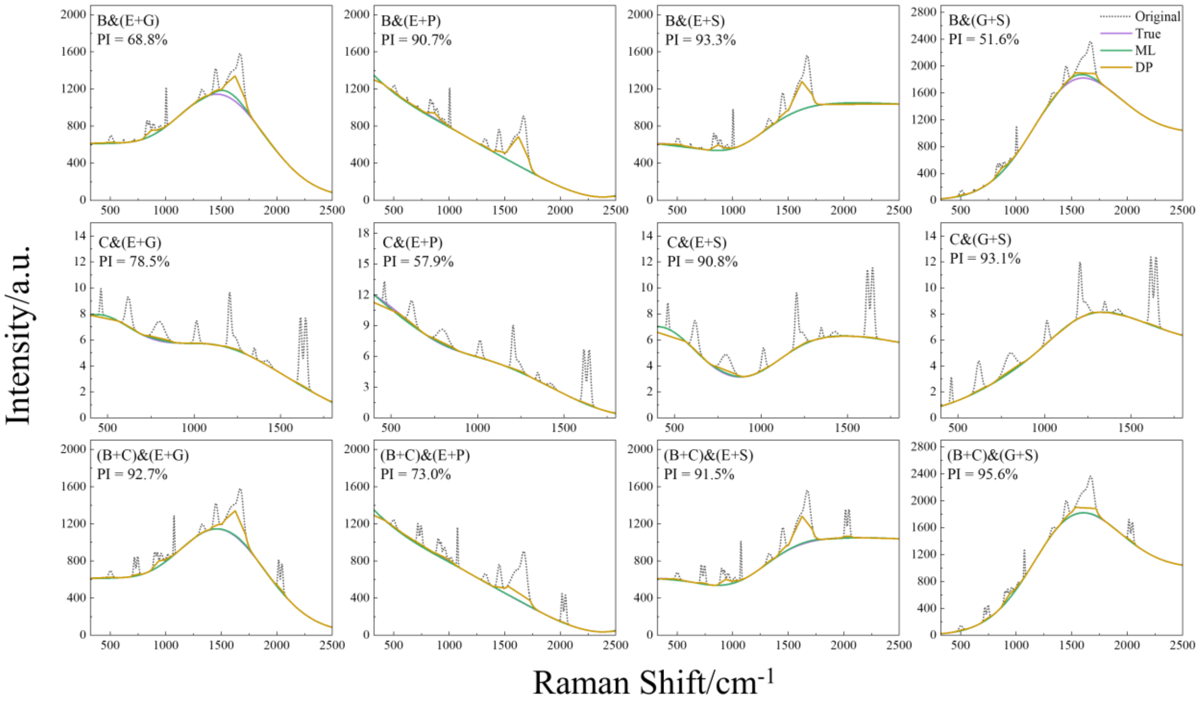

Figure S13. Performance of PCA-RF on compound spectral shapes. Rows represent peak types: broad (B), convoluted (C), and compound (B+C) peak shapes. Columns represent compound baseline combinations: (A) decreasing + decreasing (E+P), (B) decreasing + bell (E+G), (C) decreasing + increasing (E+S), and (D) bell + increasing (G+S). Each panel shows the original spectrum (black dashed line), true baseline (purple), ML-predicted baseline (green), and DP-airPLS baseline (brown). The ML model demonstrates robust performance across compound scenarios, with particularly strong results for E+S baselines and consistent performance for B+C peak shapes.

Table S9 summarizes the quantitative performance metrics for all compound spectral shapes. PCA-RF achieved PI > 50% for all 12 compound scenarios, with 7 cases exceeding PI > 90%. The E+S compound baseline showed particularly strong performance, with all peak shapes achieving PI > 90%. The B+C compound peak shape consistently met our minimum 70% PI threshold across all baseline combinations. These results confirm that ML-airPLS can handle spectral complexity that combines multiple baseline trends and peak characteristics, extending its applicability beyond the discrete categories used in model training.

Table S9. A summary of the MAE_{ML}, MAE_{DP}, and PI_{ML} values of different spectra with compound spectral shapes.

Spectrum	MAE_{ML}	MAE_{DP}	PI_{ML}
B&(E+G)	7.57	24.3	68.8%
B&(E+P)	2.59	27.6	90.7%
B&(E+S)	1.93	28.9	93.3%
B&(G+S)	7.26	15.0	51.6%
C&(E+G)	0.0110	0.0510	78.5%
C&(E+P)	0.0349	0.0831	57.9%
C&(E+S)	0.00792	0.0859	90.8%
C&(G+S)	0.00217	0.0316	93.1%
(B+C)&(E+G)	1.85	25.3	92.7%
(B+C)&(E+P)	3.70	13.7	73.0%
(B+C)&(E+S)	2.30	27.2	91.5%
(B+C)&(G+S)	0.663	16.1	95.6%

S13. Performance of ML-airPLS for noisy spectra.

We first applied ML-airPLS directly to experimental BPE and CoV229E spectra. We used WiRE software (Renishaw) as our reference standard, which provides expert-validated baseline correction algorithms and represents an established industry standard for the instrumentation used to acquire our experimental data. Then, we calculated PI values for ML-airPLS predictions.

Figure S14 illustrates representative examples comparing ML-predicted baselines (green) against WiRE reference baselines (purple) and DP-airPLS baselines (brown). For CoV229E spectra (**Figure S14A**), ML-airPLS showed modest systematic underestimation compared to WiRE baselines, particularly in peak regions (419-792, 1145-1735 cm^{-1}) and featureless regions (1841-2500 cm^{-1}), with slight overestimation only in the 823-1152 cm^{-1} region. This resulted in a PI of -6.76%, indicating minimal degradation compared to DP-airPLS performance. However, BPE spectra (**Figure S14B**) exhibited severe baseline prediction failures. ML-airPLS systematically underestimated baselines at spectral extremes (320-803 cm^{-1} and 2130-2500 cm^{-1}) while dramatically overestimating baselines, particularly in characteristic BPE peak regions (1560-1679 cm^{-1}), where the predicted baseline inappropriately followed peak contours. This resulted in a catastrophic PI of -903%, indicating that ML-airPLS increased baseline correction errors by more than nine-fold compared to DP-airPLS.

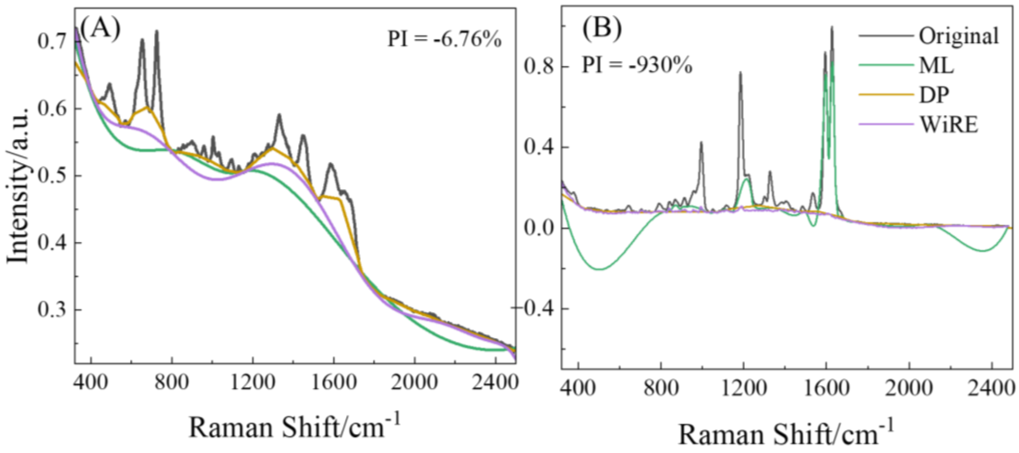

Figure S14. Baseline-correction performance of ML-airPLS on experimental SERS spectra. (A) CoV229E spectrum (black) with WiRE-derived baseline (purple), ML-optimized baseline (green), and DP baseline (brown); the ML-optimized baseline is smoother and more aligned with the WiRE baseline than the DP baseline. (B) BPE spectrum (black) with corresponding baselines; the ML baseline deviates markedly from the WiRE result, whereas the DP baseline closely follows it.

Across the complete datasets, both showed consistently negative PI. These universally negative PI values indicated that ML-airPLS baselines deviated substantially from WiRE references, with prediction errors often exceeding the baseline magnitude itself. This systematic failure motivated our investigation of noise sensitivity as a potential limiting factor. To evaluate ML-airPLS robustness under realistic experimental noise conditions, we conducted comprehensive testing on noisy synthetic spectra using systematic noise augmentation based on experimental distributions.

We defined signal-to-noise ratio (SNR) as $SNR = I_p/\sigma_{featureless}$ [27-29], where I_p represents the highest peak intensity of a given spectrum and $\sigma_{featureless}$ is the standard deviation of intensities in the featureless region (1800-2500 cm^{-1}). To calculate $\sigma_{featureless}$ for a dataset $\{I_i\}_{i=1}^{N}$, we first computed the average spectrum $\bar{I}$, then calculated $I_i' = I_i - \bar{I}$ for each spectrum, i.e., subtract each spectrum with the average. The standard deviation was computed for each spectrum's featureless region, and $\sigma_{featureless}$ was defined as the average of these individual standard deviations across all spectra.

To ensure realistic noise characteristics, we analyzed experimental SNR distributions from our BPE and CoV229E datasets, which could be fitted using a Gamma distribution with shape parameter $\alpha = 3.882$ and scale parameter $\beta = 3.884$. From this distribution, we selected 10 representative SNR levels corresponding to the 10th, 20th, ..., 90th, and 99.9th percentiles (detailed values in **Table S10**).

Table S10. A summary of the SNR percentiles and corresponding values from experimental distribution.

Index	Percentile	SNR
1	10	6.47
2	20	8.57
3	30	10.34
4	40	12.05
5	50	13.81
6	60	15.73
7	70	17.98
8	80	20.86
9	90	25.34
10	99.9	49.92

Our augmentation strategy generated noisy spectra by adding Gaussian noise $N(0, \sigma)$ to each original spectrum, where σ was calculated as the highest peak intensity divided by the target SNR. **Figure S15A** illustrates this process using a representative B&E spectrum with five different SNR levels ranging from 8.57 to 49.92. For each of the 12 spectral shapes, we augmented all 500 spectra at each of the 10 SNR levels with 5 different noise realizations, generating a total of 300,000 noisy spectra. We operated under the assumption that any augmented spectrum should have the same optimal parameters (λ^*, τ^*) as the corresponding noise-free spectrum.

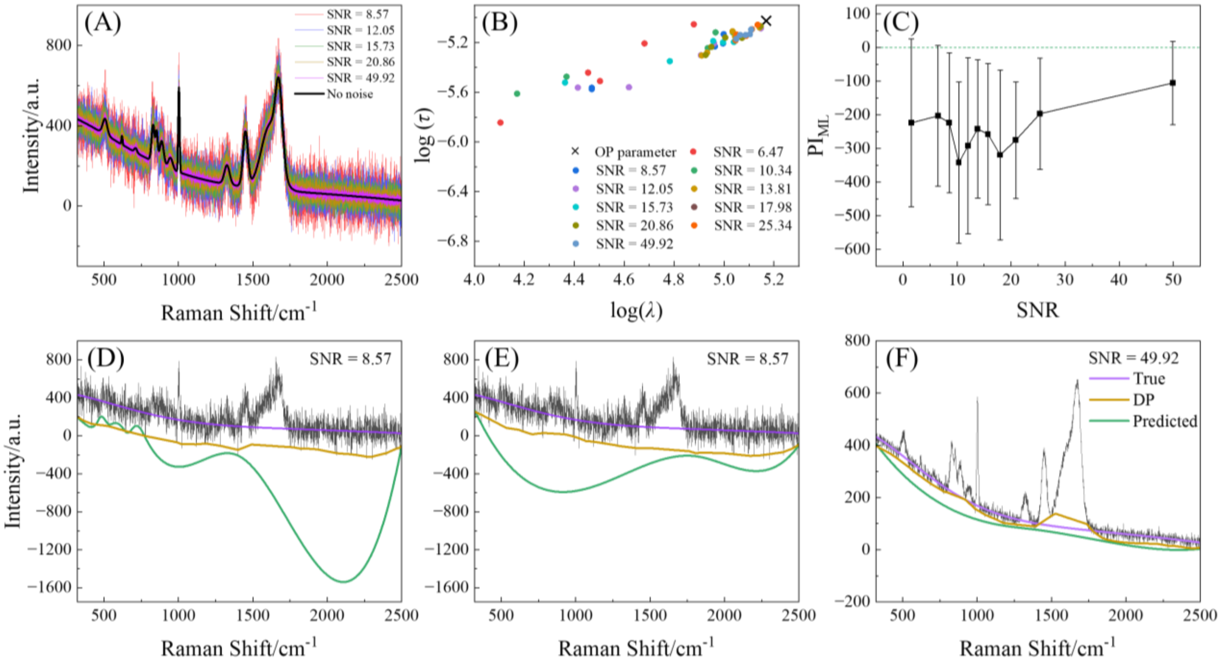

Figure S15. Performance degradation of the no-noise model across experimental SNR levels. (A) A B&E spectrum without noise (black) and its noisy counterparts at five SNR levels: 8.57 (red), 12.05 (purple), 15.73 (green), 20.86 (brown), and 49.92 (pink). (B) PCA-RF-predicted parameters for each noisy spectrum generated by the noise-free spectrum in (A), overlaid with the OP parameter (λ^*, τ^*) (black cross). (C) Average PI_{ML} with standard deviation across all spectral shapes, plotted as a function of SNR. (D) One realization of the B&E spectrum at SNR = 8.57, showing the true baseline (purple), DP baseline (brown), and ML-predicted baseline (green). (E) A different realization of the same B&E spectrum at SNR = 8.57 with corresponding baseline comparisons. (F) A realization of the B&E spectrum at SNR = 49.92, with the corresponding true, DP, and predicted baselines.

However, testing revealed systematic parameter prediction failures. **Figure S15B** demonstrates how ML-predicted parameters systematically deviate from optimal parameters as SNR decreases, with both λ and τ values consistently underestimated compared to the noise-free optimal parameters (marked by black cross). In addition, both λ and τ tends to be more underestimated with the decrease of SNR. This parameter drift translates into universally poor baseline correction performance, as shown in **Figure S15C**, where average PI values remain negative across all SNR levels for all 12 spectral shapes. Even at the highest experimental SNR (49.92), performance remains substantially below acceptable thresholds. The severity of this performance degradation is illustrated through specific examples in **Figures S15D-F**. **Figures S15D** and **E** show two different noise realizations of the same B&E spectrum at SNR = 8.57, demonstrating that even identical noise levels can produce dramatically different baseline artifacts depending on the specific random seed. The first realization exhibits a large predicted baseline dip at 1400-2500 cm^{-1}, while the second shows smaller but broader deviations from 320-1760 cm^{-1}. **Figure S15F** demonstrates that even at the highest experimental SNR (49.92), baseline predictions remain substantially underestimated compared to true baselines, confirming that noise sensitivity persists across the entire experimental SNR range.

Figure S16 provides mechanistic insight into this failure mode. **Figure S16A** shows that when optimal parameters designed for noise-free spectra are applied to noisy data (SNR = 8.57), the resulting baseline closely follows spectral noise, producing a baseline-corrected spectrum with few distinguishable features. **Figure S16B** demonstrates that even at a relatively higher SNR (49.92), optimal parameters still produce baselines that track noise rather than underlying baseline trends, resulting in poor preservation of peak intensities. This behavior proves that the $p = 2$ smoothness constraint in airPLS conflicts with noise-induced fluctuations, causing the algorithm to interpret noise as spectral features requiring baseline adjustment.

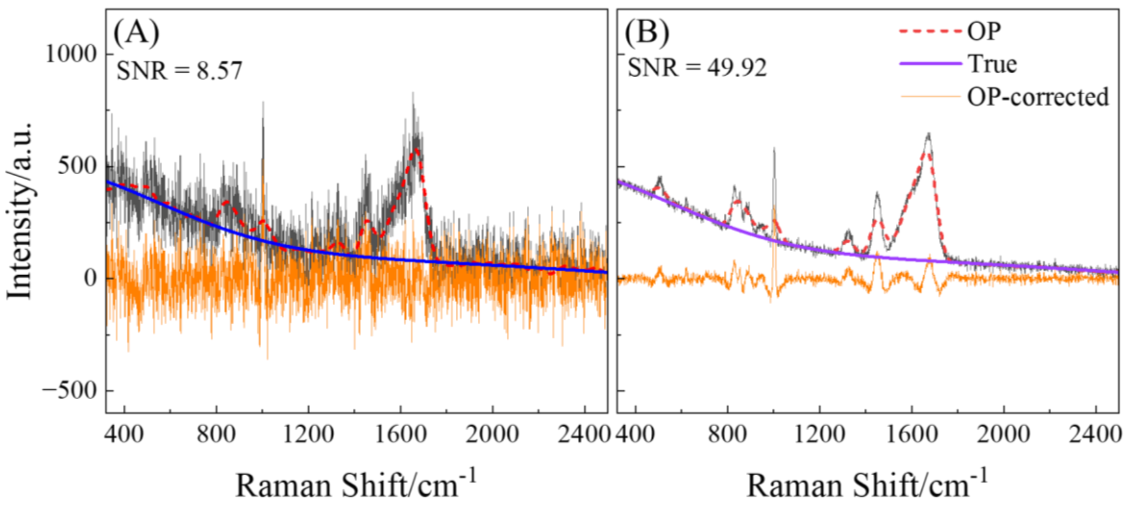

Figure S16. Failure of noise-free OP parameter on noisy spectra. (A) A representative B&E spectrum with SNR = 8.57 (black) and its true baseline (blue). The baseline predicted using the OP parameters (red dashed) closely follows the noisy spectrum, resulting in an over-corrected spectrum (orange) that lacks distinct spectral features. (B) Another example of the B&E spectrum with highest available SNR of 49.92 (black) and its true baseline (blue). Although the predicted baseline (red dashed) deviates slightly more from the spectrum, it still resembles it too closely, yielding a corrected spectrum (orange) with partially recovered features but distorted peak intensities.

S14. Performance of ML-airPLS for experimental spectra.

The results of ML-airPLS on noisy synthetic data suggest a fundamental breakpoint phenomenon: while noise-free spectra ($SNR = +\infty$) work excellently and experimental SNR levels fail, there must exist some threshold SNR above which acceptable performance can be achieved. This hypothesis motivated our exploration of denoising approaches as a preprocessing step before parameter prediction. Since the BPE and CoV229E SERS spectra exhibited SNR values within the problematic experimental range, we applied Savitzky-Golay smoothing (window length = 15, order = 2) in SpectraGuru [25] to enhance SNR to above 100. We still used WiRE software (Renishaw) as our reference standard as we did in **Section S13**.

Before evaluating ML-airPLS performance, we sought to understand why real spectra might perform differently than our synthetic training data. According to machine learning theory [26], model performance depends critically on the similarity between test data and training data distributions. Since our synthetic dataset varies primarily in baseline characteristics across the 12 spectral shapes, we hypothesized that real spectra performance would correlate with their similarity to training data. To test this hypothesis, we calculated cosine similarity between each experimental spectrum and all synthetic spectra in our training dataset. The cosine similarity between an experimental spectrum I_{exp} and a synthetic spectrum I_{syn} is defined as:

$$Cosine\ similarity(I_{exp}, I_{syn}) = \frac{I_{exp} \cdot I_{syn}}{\left\|I_{exp}\right\|_2 \left\|I_{syn}\right\|_2} \qquad (S18)$$

where $\cdot$ represents the inner product and $\| \cdot \|_2$ denotes the L^2 norm as defined in **Equation S1**. For each experimental spectrum, we computed its cosine similarity with every synthetic spectrum, then calculated the mean and standard deviation of similarity scores grouped by each of the 12 spectral shapes. Finally, we identified which spectral shape showed the highest average similarity to each experimental dataset and reported the corresponding mean and standard deviation values.

This analysis revealed important differences between datasets: CoV229E spectra showed high cosine similarity to synthetic spectra from the B&P spectral shape (0.933 ± 0.067), indicating close alignment with one of our training spectral combinations; while BPE spectra exhibited much lower similarity across all training combinations, with the highest cosine similarity to the C&E spectral shape but still poor match (0.540 ± 0.039). This quantitative spectral similarity analysis provided a predictive framework for understanding subsequent ML-airPLS performance differences. As predicted by the similarity analysis, ML-airPLS performance on real spectra varied

dramatically between datasets, as shown in **Figure 4** in the main text. The quantitative results shown in **Figure 4C** confirm this correlation: CoV229E spectra, with high spectral similarity to training data, achieved PI values ranging from approximately -40% to 67%, while BPE spectra, with poor similarity to all training combinations, consistently produced negative PI values between -70% and -3%, with some outliers having PI to even < -300.

These real-data results establish both the potential and fundamental limitations of the current ML-airPLS approach. The successful performance on CoV229E spectra demonstrates proof-of-concept feasibility when experimental baseline shapes align with training data distributions. However, the systematic failures on BPE spectra highlight two critical challenges: (1) the need for expanded training datasets covering broader baseline shape variations, and (2) the requirement for more sophisticated noise-handling strategies beyond simple denoising and parameter prediction. This comprehensive analysis provides a foundation for future developments addressing these limitations while confirming that ML-airPLS can achieve practical utility when applied within appropriate scope conditions.

Reference:

(1) Zhang, Z. M.; Chen, S.; Liang, Y. Z. Baseline correction using adaptive iteratively reweighted penalized least squares. *Analyst* **2010**, *135* (5), 1138-1146. DOI: 10.1039/b922045c.

(2) LeVeque, R. J. *Finite difference methods for ordinary and partial differential equations : steady-state and time-dependent problems*; Society for Industrial and Applied Mathematics, 2007.

(3) Lieber, C. A.; Mahadevan-Jansen, A. Automated method for subtraction of fluorescence from biological Raman spectra. *Appl Spectrosc* **2003**, *57* (11), 1363-1367. DOI: 10.1366/000370203322554518.

(4) Kumar, A.; Islam, M. R.; Zughaier, S. M.; Chen, X.; Zhao, Y. Precision classification and quantitative analysis of bacteria biomarkers via surface-enhanced Raman spectroscopy and machine learning. *Spectrochimica Acta Part A: Molecular and Biomolecular Spectroscopy* **2024**, *320*, 124627. DOI: https://doi.org/10.1016/j.saa.2024.124627.

(5) Yang, Y.; Xu, B.; Haverstick, J.; Ibtehaz, N.; Muszyński, A.; Chen, X.; Chowdhury, M. E. H.; Zughaier, S. M.; Zhao, Y. Differentiation and classification of bacterial endotoxins based on surface enhanced Raman scattering and advanced machine learning. *Nanoscale* **2022**, *14* (24), 8806-8817, 10.1039/D2NR01277D. DOI: 10.1039/D2NR01277D.

(6) Yang, Y.; Murray, J.; Haverstick, J.; Tripp, R. A.; Zhao, Y. Silver nanotriangle array based LSPR sensor for rapid coronavirus detection. *Sensors and Actuators B: Chemical* **2022**, *359*, 131604. DOI: https://doi.org/10.1016/j.snb.2022.131604.

(7) Yang, Y.; Xu, B.; Murray, J.; Haverstick, J.; Chen, X.; Tripp, R. A.; Zhao, Y. Rapid and quantitative detection of respiratory viruses using surface-enhanced Raman spectroscopy and machine learning. *Biosensors and Bioelectronics* **2022**, *217*, 114721. DOI: 10.1016/j.bios.2022.114721.

(8) Yang, Y.; Cui, J.; Luo, D.; Murray, J.; Chen, X.; Hülck, S.; Tripp, R. A.; Zhao, Y. Rapid Detection of SARS-CoV-2 Variants Using an Angiotensin-Converting Enzyme 2-Based Surface-Enhanced Raman Spectroscopy Sensor Enhanced by CoVari Deep Learning Algorithms. *ACS Sensors* **2024**, *9* (6), 3158-3169. DOI: 10.1021/acssensors.4c00488.

(9) Yang, Y.; Cui, J.; Kumar, A.; Luo, D.; Murray, J.; Jones, L.; Chen, X.; Hülck, S.; Tripp, R. A.; Zhao, Y. Multiplex Detection and Quantification of Virus Co-Infections Using Label-free Surface-Enhanced Raman Spectroscopy and Deep Learning Algorithms. *ACS Sensors* **2025**, *10* (2), 1298-1311. DOI: 10.1021/acssensors.4c03209.

(10) Zhao, Y.; Kumar, A.; Yang, Y. Unveiling practical considerations for reliable and standardized SERS measurements: lessons from a comprehensive review of oblique angle deposition-fabricated silver nanorod array substrates. *Chemical Society Reviews* **2024**, *53* (2), 1004-1057, 10.1039/D3CS00540B. DOI: 10.1039/D3CS00540B.

(11) Ye, J.; Tian, Z.; Wei, H.; Li, Y. Baseline correction method based on improved asymmetrically reweighted penalized least squares for the Raman spectrum. *Appl Opt* **2020**, *59* (34), 10933-10943. DOI: 10.1364/AO.404863.

(12) Chen, K.; Zhang, H.; Wei, H.; Li, Y. Improved Savitzky-Golay-method-based fluorescence subtraction algorithm for rapid recovery of Raman spectra. *Appl Opt* **2014**, *53* (24), 5559-5569. DOI: 10.1364/AO.53.005559.

(13) Schulze, H. G.; Foist, R. B.; Okuda, K.; Ivanov, A.; Turner, R. F. A small-window moving average-based fully automated baseline estimation method for Raman spectra. *Appl Spectrosc* **2012**, *66* (7), 757-764. DOI: 10.1366/11-06550.

(14) Xi, Y.; Li, Y.; Duan, Z.; Lu, Y. A Novel Pre-Processing Algorithm Based on the Wavelet Transform for Raman Spectrum. *Applied Spectroscopy* **2018**, *72* (12), 1752-1763. DOI: 10.1177/0003702818789695.
(15) He, S.; Zhang, W.; Liu, L.; Huang, Y.; He, J.; Xie, W.; Wu, P.; Du, C. Baseline correction for Raman spectra using an improved asymmetric least squares method. *Anal. Methods* **2014**, *6* (12), 4402-4407. DOI: 10.1039/c4ay00068d.
(16) Liu, J.; Sun, J.; Huang, X.; Li, G.; Liu, B. Goldindec: A Novel Algorithm for Raman Spectrum Baseline Correction. *Appl Spectrosc* **2015**, *69* (7), 834-842. DOI: 10.1366/14-07798.
(17) Chen, H.; Xu, W.; Broderick, N. G. R. An Adaptive and Fully Automated Baseline Correction Method for Raman Spectroscopy Based on Morphological Operations and Mollification. *Appl Spectrosc* **2019**, *73* (3), 284-293. DOI: 10.1177/0003702818811688.
(18) Ning, X. R.; Selesnick, I. W.; Duval, L. Chromatogram baseline estimation and denoising using sparsity (BEADS). *Chemometrics and Intelligent Laboratory Systems* **2014**, *139*, 156-167. DOI: 10.1016/j.chemolab.2014.09.014.
(19) Zhao, J.; Lui, H.; McLean, D. I.; Zeng, H. Automated Autofluorescence Background Subtraction Algorithm for Biomedical Raman Spectroscopy. *Applied Spectroscopy* **2007**, *61* (11), 1225-1232. DOI: 10.1366/000370207782597003.
(20) Breiman, L. Random forests. *Mach Learn* **2001**, *45* (1), 5-32. DOI: Doi 10.1023/A:1010933404324.
(21) Chen, T.; Guestrin, C. XGBoost: A Scalable Tree Boosting System. *Proceedings of the 22nd ACM SIGKDD International Conference on Knowledge Discovery and Data Mining* **2016**, 785–794. DOI: 10.1145/2939672.2939785.
(22) Hochreiter, S.; Schmidhuber, J. Long Short-Term Memory. *Neural Computation* **1997**, *9* (8), 1735-1780. DOI: 10.1162/neco.1997.9.8.1735.
(23) LeCun, Y.; Bengio, Y.; Hinton, G. Deep learning. *Nature* **2015**, *521* (7553), 436-444. DOI: 10.1038/nature14539.
(24) He, K.; Zhang, X.; Ren, S.; Sun, J. Deep Residual Learning for Image Recognition. *2016 IEEE Conference on Computer Vision and Pattern Recognition (CVPR)* **2015**, 770-778.
(25) *SpectraGuru*. https://www.zhao-nano-lab.com/spectraguru (accessed 2025 Feb 13).
(26) Gulrajani, I.; Lopez-Paz, D. In Search of Lost Domain Generalization. 2020; p arXiv:2007.01434.
(27) Harvey, T. J.; Hughes, C.; Ward, A. D.; Faria, E. C.; Henderson, A.; Clarke, N. W.; Brown, M. D.; Snook, R. D.; Gardner, P. Classification of fixed urological cells using Raman tweezers. *Journal of Biophotonics* **2009**, *2* (1-2), 47-69. DOI: https://doi.org/10.1002/jbio.200810061 (acccessed 2025/06/13).
(28) Barton, S. J.; Ward, T. E.; Hennelly, Bryan M. Algorithm for optimal denoising of Raman spectra. *Analytical Methods* **2018**, *10* (30), 3759-3769, 10.1039/C8AY01089G. DOI: 10.1039/C8AY01089G.
(29) Schulze, H. G.; Yu, M. M. L.; Addison, C. J.; Blades, M. W.; Turner, R. F. B. Automated Estimation of White Gaussian Noise Level in a Spectrum with or without Spike Noise Using a Spectral Shifting Technique. *Applied Spectroscopy* **2006**, *60* (7), 820-825. DOI: 10.1366/000370206777887134 (acccessed 2025/06/13).
